# A bispecific T cell engager recruits both type 1 NKT and Vγ9Vδ2-T cells for the treatment of CD1d-expressing hematological malignancies

**DOI:** 10.1016/j.xcrm.2023.100961

**Published:** 2023-03-02

**Authors:** Roeland Lameris, Jurjen M. Ruben, Victoria Iglesias-Guimarais, Milon de Jong, Myrthe Veth, Fleur S. van de Bovenkamp, Iris de Weerdt, Arnon P. Kater, Sonja Zweegman, Sjeng Horbach, Thilo Riedl, Benjamin Winograd, Rob C. Roovers, Anton E.P. Adang, Tanja D. de Gruijl, Paul W.H.I. Parren, Hans J. van der Vliet

**Affiliations:** 1 Amsterdam UMC location Vrije University Amsterdam, Department of Medical Oncology, Cancer Center Amsterdam, Amsterdam, the Netherlands; 2 LAVA Therapeutics, Utrecht, the Netherlands; 3 Amsterdam UMC location University of Amsterdam, Department of Hematology, Cancer Center Amsterdam, Amsterdam, the Netherlands; 4 Amsterdam UMC location Vrije University Amsterdam, Department of Hematology, Cancer Center Amsterdam, Amsterdam, the Netherlands; 5 J&S Preclinical Solutions, Oss, the Netherlands; 6 LAVA Therapeutics, Philadelphia, PA, USA; 7 Leiden University Medical Center, Department of Immunology, Leiden, the Netherlands

**Keywords:** bispecific T cell engagers, single-domain antibody, CD1d, Vγ9Vδ2-T cell, type 1 NKT cell, multiple myeloma, acute myeloid leukemia, chronic lymphocytic leukemia, preclinical, non-human primate

## Abstract

Bispecific T cell engagers (bsTCEs) hold great promise for cancer treatment but face challenges due to the induction of cytokine release syndrome (CRS), on-target off-tumor toxicity, and the engagement of immunosuppressive regulatory T cells that limit efficacy. The development of Vγ9Vδ2-T cell engagers may overcome these challenges by combining high therapeutic efficacy with limited toxicity. By linking a CD1d-specific single-domain antibody (VHH) to a Vδ2-TCR-specific VHH, we create a bsTCE with trispecific properties, which engages not only Vγ9Vδ2-T cells but also type 1 NKT cells to CD1d^+^ tumors and triggers robust proinflammatory cytokine production, effector cell expansion, and target cell lysis *in vitro*. We show that CD1d is expressed by the majority of patient MM, (myelo)monocytic AML, and CLL cells and that the bsTCE triggers type 1 NKT and Vγ9Vδ2-T cell-mediated antitumor activity against these patient tumor cells and improves survival in *in vivo* AML, MM, and T-ALL mouse models. Evaluation of a surrogate CD1d-γδ bsTCE in NHPs shows Vγ9Vδ2-T cell engagement and excellent tolerability. Based on these results, CD1d-Vδ2 bsTCE (LAVA-051) is now evaluated in a phase 1/2a study in patients with therapy refractory CLL, MM, or AML.

## Highlights

• CD1d is expressed on the majority of patient MM, (myelo)monocytic AML, and CLL cells
• A CD1d-Vδ2 bsTCE triggers dual activation of type 1 NKT and Vγ9Vδ2-T cells
• CD1d-Vδ2 bsTCE-activated type 1 NKT and Vγ9Vδ2-T cells exert robust antitumor activity
• A surrogate fully cross-reactive CD1d-γδ bsTCE is well tolerated in NHP studies

## Graphical abstract

**Figure:**
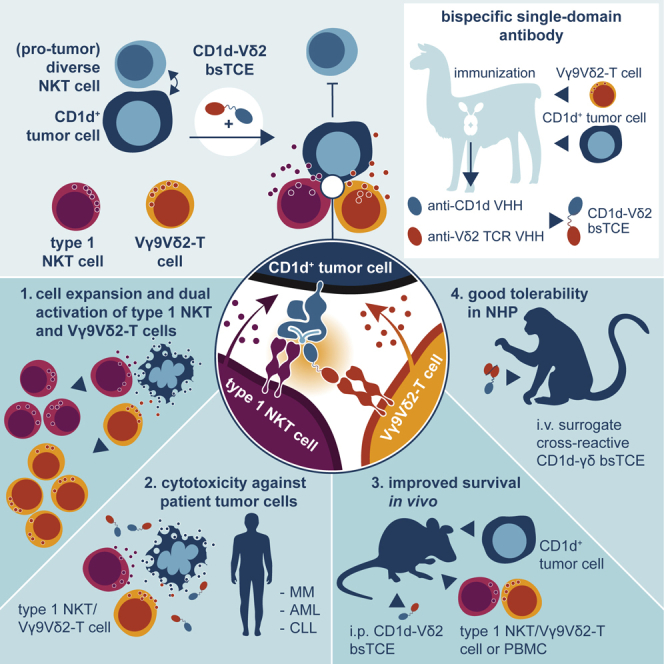

## Teaser

Bispecific T cell engagers (bsTCEs) show anticancer therapeutic activity but face challenges due to toxicity and regulatory T cell engagement, limiting efficacy. Lameris et al. demonstrate that selective engagement of Vγ9Vδ2-T and type 1 NKT cells using a CD1d-Vδ2 bsTCE results in robust antitumor activity and is well tolerated in NHPs.

## Introduction

T cell-engaging therapies are promising approaches in an expanding number of malignancies.^1^ Bispecific T cell engagers (bsTCEs) offer “off-the-shelf” immunotherapies that induce signaling most commonly via the CD3-T cell receptor (TCR) complex upon binding to a second target protein on tumor cells, thereby triggering T cell-mediated tumor lysis.^1^^,^^2^ In particular in malignancies of B cell origin, impressive clinical results have been obtained with CD3-targeting bsTCEs (e.g., blinatumomab), but toxicities such as cytokine release syndrome (CRS) and immune effector cell-associated neurotoxicity syndrome (ICANS) often occur, and broadening of the use of bsTCE, specifically to solid tumors, has been challenging.^1^^,^^2^^,^^3^^,^^4^ Indeed, to mitigate toxicity, priming doses and corticosteroid pre-medication, which might negatively affect clinical outcome, are often used.^1^^,^^5^ Moreover, concomitant engagement of regulatory T cells (Tregs) by CD3-targeting bsTCEs is known to negatively affect clinical outcome and can even interfere with antitumor activity in patients treated with blinatumomab.^6^^,^^7^ Engaging innate-like T cell subsets with inherent antitumor activity, such as type 1 natural killer T (NKT) and Vγ9Vδ2-T cells, could combine high therapeutic efficacy with a reduced risk of CRS and off-tumor toxicity.

Vγ9Vδ2-T cells represent a sizable (1%–10% of T cells) and homogeneous immune effector T cell population that responds to intracellular accumulation of phosphoantigens (pAgs) by sensing conformational changes in the butyrophilin (BTN)2A1-BTN3A1 complex.^8^ Endogenous pAgs are metabolites of the mevalonate pathway and frequently accumulate in malignant cells as a result of metabolic dysregulation,^9^ which sensitizes these cells for Vγ9Vδ2-T cell-mediated lysis.^10^^,^^11^ Upon stimulation, Vγ9Vδ2-T cells rapidly produce T helper (T_h_) 1-type cytokines, exert a direct cytotoxic effect via perforin/granzyme B and Fas ligand, and can (cross-)present antigens (Ags) in a professional co-stimulatory context.^10^^,^^11^^,^^12^ Both peripheral blood and tumor-infiltrating Vγ9Vδ2-T cell content positively correlate with clinical outcome in several malignancies.^10^^,^^11^^,^^13^^,^^14^ While therapies using either pAg-based approaches alone or in combination with low-dose interleukin (IL)-2 and/or adoptive transfer of *ex vivo* expanded Vγ9Vδ2-T cells were safe and induced objective antitumor responses in several patients, the overall induction of clinical responses was modest.^11^^,^^15^ One can envision that tumor-targeted activation of Vγ9Vδ2-T cells, an aspect that was lacking in previous studies, could significantly improve the consistency and robustness of Vγ9Vδ2-T cell-directed therapies. Indeed, we and others have shown successful engagement of Vγ9Vδ2-T cells via a bsTCE that crosslinks an epitope on either the TCR Vγ9 or Vδ2 chain with an epitope on a tumor-associated Ag, demonstrating antitumor activity in multiple preclinical models.^16^^,^^17^^,^^18^^,^^19^^,^^20^

Type 1 NKT cells, which express a semi-invariant TCR and respond to both self and foreign (glyco)lipid Ags presented in the context of the non-polymorphic major histocompatibility complex (MHC) class I-like molecule CD1d, have well-documented antitumor properties.^10^^,^^21^^,^^22^ Upon activation, this relatively rare (∼0.1% of total T cells) but powerful T cell subset has multimodal activity via bidirectional crosstalk with Ag-presenting cells (e.g., B cells, dendritic cells [DCs]) inducing mixed T_h_1/T_h_2-type cytokines triggering downstream effector cell activation, and direct lysis of CD1d^+^ tumor cells, tumor-associated macrophages (TAMs), and myeloid-derived suppressor cells (MDSCs) via perforin/granzyme B and Fas ligand cytotoxic pathways.^10^^,^^21^^,^^22^ While therapies targeting the CD1d-type 1 NKT cell axis using glycolipid Ag-based approaches and/or adoptive transfer of type 1 NKT cells were proven safe and could induce objective antitumor responses, the overall response rate was insufficient in patients.^10^^,^^21^^,^^22^

Recently, we reported on the identification of a camelid-derived single-domain antibody (VHH1D12) that triggers robust type 1 NKT cell activation. By using a functional and structural approach, we showed that VHH1D12 simultaneously contacts CD1d and the type 1 NKT TCR, thereby stabilizing this interaction through intrinsic bispecificity, which translates directly into antitumor activity and has the potential to overcome at least some of the clinically encountered limitations of glycolipid Ag-based therapeutic approaches. Indeed, a superior effect of VHH1D12 over glycolipid Ag on type 1 NKT cell-mediated antitumor activity was noted using patient-derived tumor samples.^23^

Here, we generated a CD1d-Vδ2 bsTCE that can trigger robust activation of both type 1 NKT and Vγ9Vδ2-T cells toward CD1d^+^ tumor cells, resulting in strong activity in *in vivo* models and *ex vivo* against patient multiple myeloma (MM), monocytic acute myeloid leukemia (AML), and chronic lymphocytic leukemia (CLL) cells. Exploratory toxicology studies with a fully cross-reactive (surrogate) CD1d-γδ bsTCE in non-human primates (NHPs) demonstrated good tolerability, no clinical, chemistry, or hematology abnormalities or organ toxicity, and only low levels of IL-6.

## Results

### Dual type 1 NKT cell and Vγ9Vδ2-T cell activation and antitumor activity by CD1d-Vδ2 bsTCE

**Figure 1:**
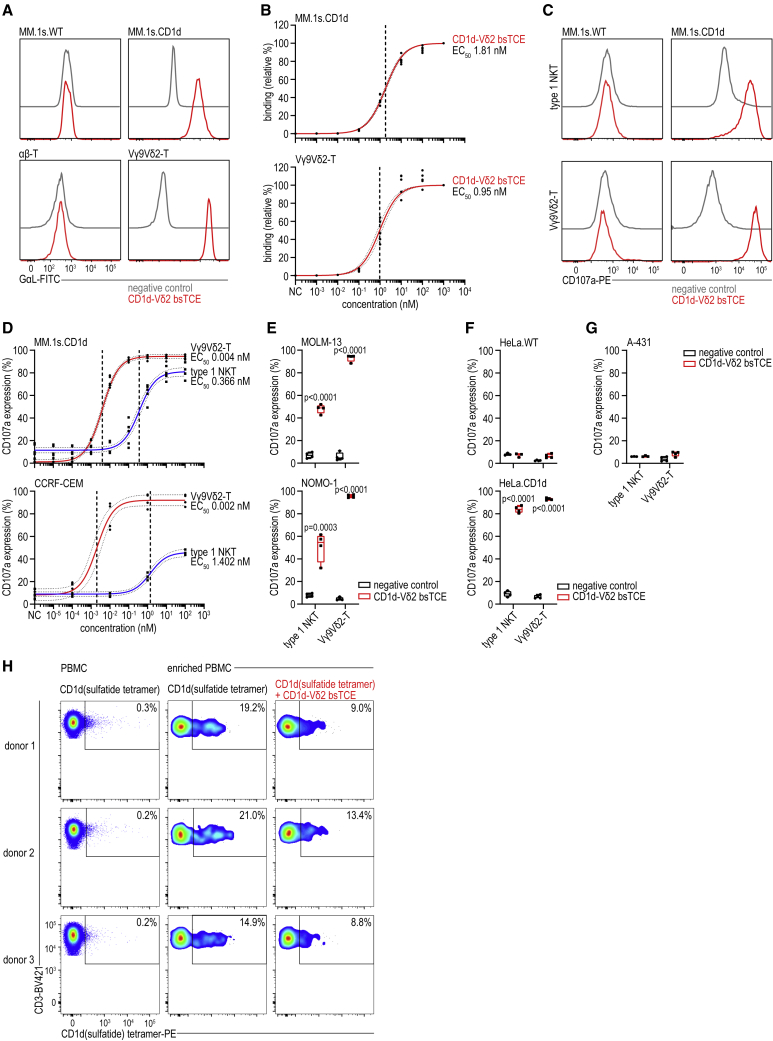
CD1d-Vδ2 bsTCE activates both type 1 NKT and Vγ9Vδ2-T cells (A and B) Relative binding of CD1d-Vδ2 bsTCE to MM.1s.wild-type (WT) (n = 3), MM.1s.CD1d (n = 5), αβ-T (n = 3), or Vγ9Vδ2-T cells (n = 5), detected by goat-anti-llama (GαL)-FITC. A representative histogram at 1,000 nM (A) or a concentration range (B) is shown. (C–G) CD107a expression on type 1 NKT and Vγ9Vδ2-T cells after 4-h co-culture with either CD1d^−^ MM.1s.WT (C, representative histograms), HeLa.WT (F, type 1 NKT n = 3, Vγ9Vδ2-T n = 4) or A-431 (G, type 1 NKT n = 3, Vγ9Vδ2-T n = 4) or CD1d^+^ MM.1s.CD1d (C, representative histograms and D, n = 5), CCRF-CEM (D, n = 4), MOLM-13 (E, n = 4), NOMO-1 (E, n = 4), or HeLa.CD1d cells (F, n = 4) ± 100 nM (C, E, F) or concentration range (D) of CD1d-Vδ2 bsTCE. (H) Flow cytometry plots of three donors depicting the binding of CD1d(sulfatide) tetramers pre-incubated ± 2 μM CD1d-Vδ2 bsTCE to PBMC or PBMC enriched for sulfatide-reactive diverse NKT cells using tetramer-associated magnetic enrichment. The number in each plot indicates the percentage of CD1d(sulfatide) tetramer-PE^+^ cells (of CD3^+^ cells). Negative control (NC) indicates PBS (A and B) or medium control (C–G). Box-and-whisker plots indicate the median, 25th–75th percentiles, and minimum-maximum. Non-linear regression with 95% confidence bands (dotted lines) and EC_50s_ (dashed lines) (B and D). Two-way ANOVA with Šídák multiple comparisons test (E and F). See also figure S1.

The CD1d-Vδ2 bsTCE (molecular weight [MW] ∼27 kDa; Table S1), generated by fusing the CD1d-specific VHH1D12 with the Vδ2-TCR-specific VHH5C8, was designed to engage and conditionally activate both type 1 NKT and Vγ9Vδ2-T cells upon binding to CD1d. Binding experiments showed that the specificity and half-maximal effective concentration (EC_50_) of the CD1d-Vδ2 bsTCE for CD1d and Vγ9Vδ2-TCR was in the low-nanomolar range and equivalent to that of the individual monospecific VHHs (Figures 1A, 1B, and S1A). The CD1d-Vδ2 bsTCE triggered robust upregulation of the degranulation marker CD107a on both type 1 NKT and Vγ9Vδ2-T cells in co-culture with multiple CD1d^+^ (but not CD1d^−^) tumor cell lines with low-nanomolar and low-picomolar EC_50s_, respectively (Figures 1C–1G). Equally low EC_50s_ were observed in co-cultures of CD1d^+^ tumor cells and mixed (1:1) type 1 NKT/Vγ9Vδ2-T cells (Figures S1B–S1D). Finally, the ability of VHH1D12 to activate type 1 NKT cells (Figure S1E) and to block diverse NKT cells (which interact with a different epitope on CD1d, adopt an alternative docking mode over CD1d, and include pro-tumor sulfatide-reactive type 2 NKT cells^22^) was preserved. The capacity of the CD1d-Vδ2 bsTCE to abrogate binding and reactivity of diverse NKT cells was shown using the sulfatide-restricted NKT cell line JRT3.DP10.7 and additionally confirmed using healthy donor peripheral blood mononuclear cell (PBMC) fractions that were enriched for sulfatide-reactive diverse NKT cells (Figures 1H, S1F, and S1G).

**Figure 2:**
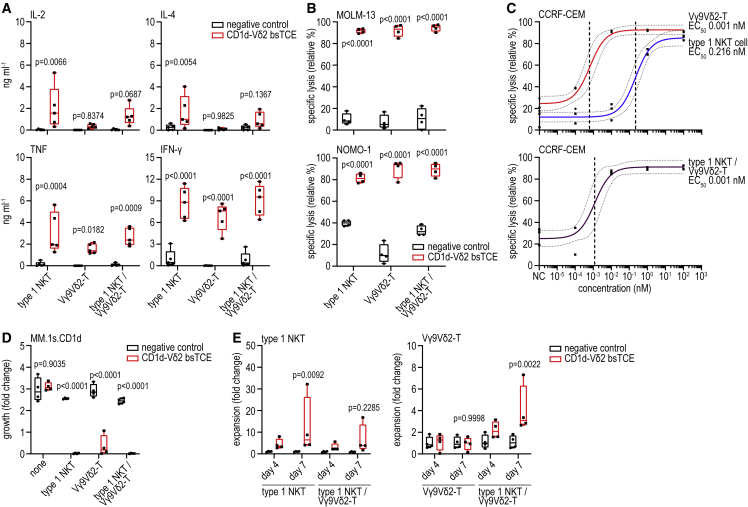
CD1d-Vδ2 bsTCE triggers both type 1 NKT and Vγ9Vδ2-T cell cytokine production, expansion, and lysis of CD1d-expressing target cells (A) Cytokine secretion by type 1 NKT, Vγ9Vδ2-T cells or a 1:1 mixture thereof after 24-h co-culture with MM.1s.CD1d cells ± 50 nM CD1d-Vδ2 bsTCE (n = 5). (B and C) Specific lysis of MOLM-13 (B, n = 4), NOMO-1 (B, n = 4), and CCRF-CEM (C, n = 3) cells after 16-h co-culture with type 1 NKT, Vγ9Vδ2-T cells, or a 1:1 mixture thereof (E:T ratio 1:2) ± 100 nM (B) or concentration range (C) of CD1d-Vδ2 bsTCE. (D and E) Fold growth of MM.1s.CD1d cells (D, n = 4, day 7) and fold expansion of type 1 NKT and Vγ9Vδ2-T cells (E, alone or together in a 2:3 type 1 NKT/Vγ9Vδ2-T cell ratio, n = 4) after 4–7 days (co-)culture (E:T ratio of 1:10) ± 50 nM CD1d-Vδ2 bsTCE. NC indicates medium control. Box-and-whisker plots indicate the median, 25th–75th percentiles, and minimum-maximum. Non-linear regression with 95% confidence bands (dotted lines) and EC_50_ (dashed lines) (C). Two-way ANOVA with Šídák multiple comparisons test (A, B, D, and E). See also Figure S2.

In co-cultures with CD1d^+^ tumor cell lines, the CD1d-Vδ2 bsTCE triggered type 1 NKT cells to secrete T_h_1 and T_h_2-type cytokines, including IL-2, IL-4, tumor necrosis factor (TNF), and interferon (IFN)-γ, whereas Vγ9Vδ2-T cells mainly secreted TNF and IFN-γ (Figure 2A). IL-6 and IL-17 were not detectable, and IL-10 levels did not increase upon CD1d-Vδ2 bsTCE stimulation (Figure S2A). IFN-γ levels in co-cultures containing both type 1 NKT and Vγ9Vδ2-T cells were higher than the sum produced by either population alone (Figure S2B).

CD1d-Vδ2 bsTCE triggered near-complete lysis of AML cell lines in co-cultures with type 1 NKT, Vγ9Vδ2-T cells, or a mixture thereof (E:T ratio 1:2) (Figures 2B and S2C). Similar cytotoxic activity was observed in co-cultures with a T cell acute lymphoblastic leukemia (T-ALL) cell line, with an EC_50_ in the sub-nanomolar range for type 1 NKT and in the low-picomolar range for Vγ9Vδ2-T cells and a mixture (1:1) of type 1 NKT/Vγ9Vδ2-T cells (Figure 2C). Moreover, in 7-day co-cultures of CD1d^+^ tumor cell lines and type 1 NKT and/or Vγ9Vδ2-T cells, CD1d-Vδ2 bsTCE mediated antitumor activity at a low E:T ratio of 1:10, which was variably accompanied by expansion of type 1 NKT and/or Vγ9Vδ2-T cells (Figures 2D, 2E, S2D, and S2E).

**Figure 3:**
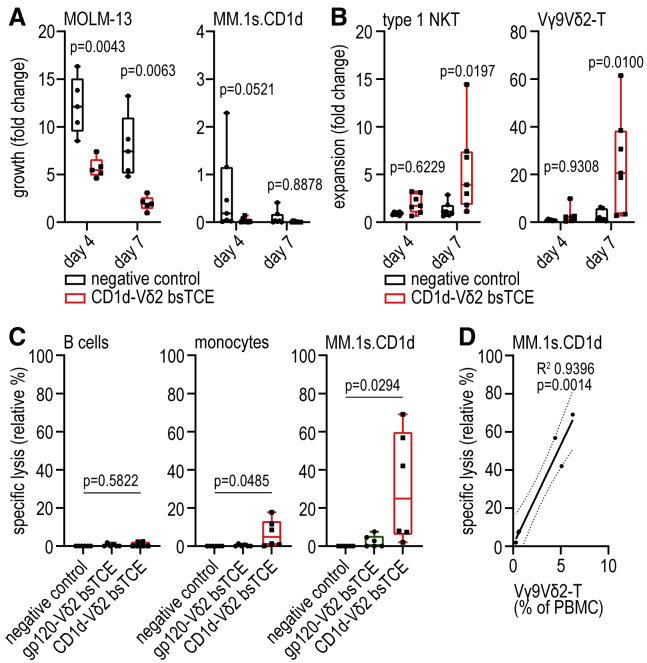
CD1d-Vδ2 bsTCE-induced lysis of CD1d+ target cells is accompanied by expansion of type 1 NKT and Vγ9Vδ2-T cells in healthy donor PBMCs (A) Fold growth of MOLM-13 (n = 5) and MM.1s.CD1d (n = 7) cells after 4–7 days’ co-culture with PBMCs (PBMC:tumor ratio 10:1, mean type 1 NKT:tumor ratio 1:856 [range 10–3,046], and mean Vγ9Vδ2-T:tumor ratio 1:17 [range 5–50]) ± 50 nM CD1d-Vδ2 bsTCE. (B) Fold expansion of type 1 NKT and Vγ9Vδ2-T cells after 4–7 days’ co-culture of PBMC and MM.1s.CD1d cells (ratio 10:1) ± 50 nM CD1d-Vδ2 bsTCE (n = 7). (C and D) Specific lysis of B cells, monocytes, and MM.1s.CD1d cells (C) and correlation of PBMC Vγ9Vδ2-T cell frequency with specific lysis of MM.1s.CD1d (D) after a 16-h co-culture of PBMCs and MM.1s.CD1d cells (ratio 10:1) ± 50 nM bsTCE (n = 6). Negative control indicates medium control. Box-and-whisker plots indicate median, 25th–75th percentiles, and minimum-maximum. Two-way ANOVA with Šídák multiple comparisons test (A and B), one-way ANOVA with Tukey multiple comparison test (C), linear regression with 95% confidence bands (dotted lines) (D). See also Figure S2.

Importantly, in 7-day co-cultures of healthy donor-derived PBMCs (i.e., non-enriched, non-preactivated type 1 NKT and Vγ9Vδ2-T cells) and tumor cell lines, AML growth was controlled and MM growth further reduced in the presence of the CD1d-Vδ2 bsTCE even at low to very low E:T ratios (Figure 3A). Particularly in the presence of MM cells, CD1d-Vδ2 bsTCE promoted strong type 1 NKT and Vγ9Vδ2-T cell expansion even in the absence of exogenously added cytokines (Figures 3B and S2F–S2H). As (non-malignant) B cells and monocytes express CD1d (Figure S2I), targeting type 1 NKT and Vγ9Vδ2-T cells to CD1d has a potential risk of on-target off-tumor toxicity. In co-cultures of PBMCs and MM cells, the cytotoxic response induced by CD1d-Vδ2 bsTCE, which correlated with the Vγ9Vδ2-T cell frequency, was skewed toward malignant cells with only limited cytotoxicity toward autologous monocytes and B cells (Figures 3C and 3D). In co-cultures of purified (untouched) Vγ9Vδ2-T cells with either the AML tumor cell line THP-1 or purified (untouched) monocytes (expressing similar levels of CD1d), the CD1d-Vδ2 bsTCE triggered Vγ9Vδ2-T cells to lyse THP-1 cells but not monocytes (Figures S2I and S2J).

Collectively, these data demonstrate that CD1d-Vδ2 bsTCEs trigger dual engagement of type 1 NKT and Vγ9Vδ2-T cells, which results in their expansion and potent and selective CD1d-restricted antitumor activity.

### CD1d-Vδ2 bsTCE humanization

**Figure 4:**
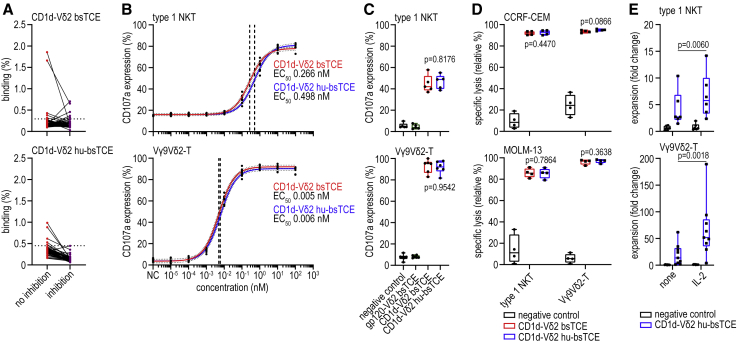
Humanization of CD1d-Vδ2 bsTCE reduces binding of pre-existing anti-VHH antibodies without affecting functionality (A) Binding of pre-existing human anti-VHH antibodies to I^125^-labeled CD1d-Vδ2 bsTCE and CD1d-Vδ2 hu-bsTCE whether or not in the presence of ∼200 times excess unlabeled bsTCE (inhibition) (n = 50). (B) CD107a expression on type 1 NKT and Vγ9Vδ2-T cells after 4-h co-culture with MM.1s.CD1d cells ± concentration range of indicated bsTCE (n = 4). (C) CD107a expression on type 1 NKT and Vγ9Vδ2-T cells after a 16-h co-culture of PBMCs and MM.1s.CD1d cells (ratio 10:1) ± 50 nM indicated bsTCE (type 1 NKT n = 5, Vγ9Vδ2-T n = 6). (D) Specific lysis of CCRF-CEM and MOLM-13 cells after 16-h co-culture with type 1 NKT or Vγ9Vδ2-T cells (E:T ratio 1:2) ± 50 nM indicated bsTCE (n = 4). (E) Fold expansion of type 1 NKT (n = 6) and Vγ9Vδ2-T cells (n = 9) after 7 days’ co-culture of PBMC and MM.1s.CD1d cells (ratio 10:1) ± 10 nM CD1d-Vδ2 bsTCE ± rhIL-2 (100 IU mL^−1^). NC indicates medium control. Box-and-whisker plots indicate median, 25th–75th percentiles, and minimum-maximum. Non-linear regression with 95% confidence bands (dotted lines) and EC_50s_ (dashed lines) (B). One-way ANOVA with Tukey multiple comparisons test (C), and two-way ANOVA with Šídák multiple comparisons test (D and E). See also Figure S3.

VHH have a reported low intrinsic immunogenicity risk profile due to their small size, high stability, rapid blood clearance, and high degree of sequence homology with the human immunoglobulin heavy-chain variable domain (IGHV).^24^^,^^25^ Indeed, compared with the most similar human germline variable heavy-chain gene segment, CD1d VHH1D12 and Vδ2 VHH5C8 had a respective 76.3% and 79.6% identity (Table S2). Nevertheless, pre-existing and infrequently occurring treatment-induced anti-drug antibodies (ADAs) have been reported, and toxicities, possibly related to ADA, have been described in clinical trials that evaluated VHH-based therapies.^24^^,^^26^^,^^27^^,^^28^^,^^29^ To further minimize immunogenicity and prevent development and/or binding of pre-existing (potentially neutralizing) ADAs, 10 humanized sequence variants of CD1d VHH1D12 and Vδ2 VHH5C8 were generated and their human sequence identity and immunogenicity risk (HLA-DRB1-binding) scores were calculated (STAR Methods; Tables S1 and S2). All humanized CD1d variants had reduced CD1d binding and triggered reduced, although more variable, type 1 NKT cell degranulation (Figures S3A and S3B). Vγ9Vδ2-TCR binding of all humanized Vδ2 VHH variants was unaffected (Figures S3C and S3D). Anti-Vδ2 VHH variant 1 was selected as the most favorable humanized candidate (STAR Methods; Table S2) and linked to the C terminus of anti-CD1d VHH1D12 with only a Q1E substitution (this first amino acid does not interact with CD1d^23^ and was substituted to increase leader peptide cleavage efficiency in the *Pichia pastoris* yeast strain selected for large-scale production). The humanized CD1d-Vδ2 (hu-)bsTCE had an immunogenicity risk in the range of humanized antibodies. When tested against sera from healthy human donors, the hu-bsTCE showed a low incidence and level of binding to pre-existing human anti-VHH antibodies (Figure 4A). Binding EC_50_ for both CD1d and Vγ9Vδ2-T cells and *in vitro* functional activity (degranulation and tumor lysis) was identical between humanized and wild-type (WT) CD1d-Vδ2 bsTCE (Figures 4B–4D, S3E, and S3F). Similar to the CD1d-Vδ2 bsTCE, the hu-bsTCE promoted expansion of type 1 NKT and Vγ9Vδ2-T cells, and this could be further boosted by exogenous IL-2 (Figure 4E).

In conclusion, CD1d-Vδ2 bsTCE humanization was successful and reduced the predicted immunogenicity risk without compromising functionality.

### CD1d-Vδ2 bsTCE triggers antitumor activity against patient-derived MM, monocytic AML, and CLL cells

**Figure 5:**
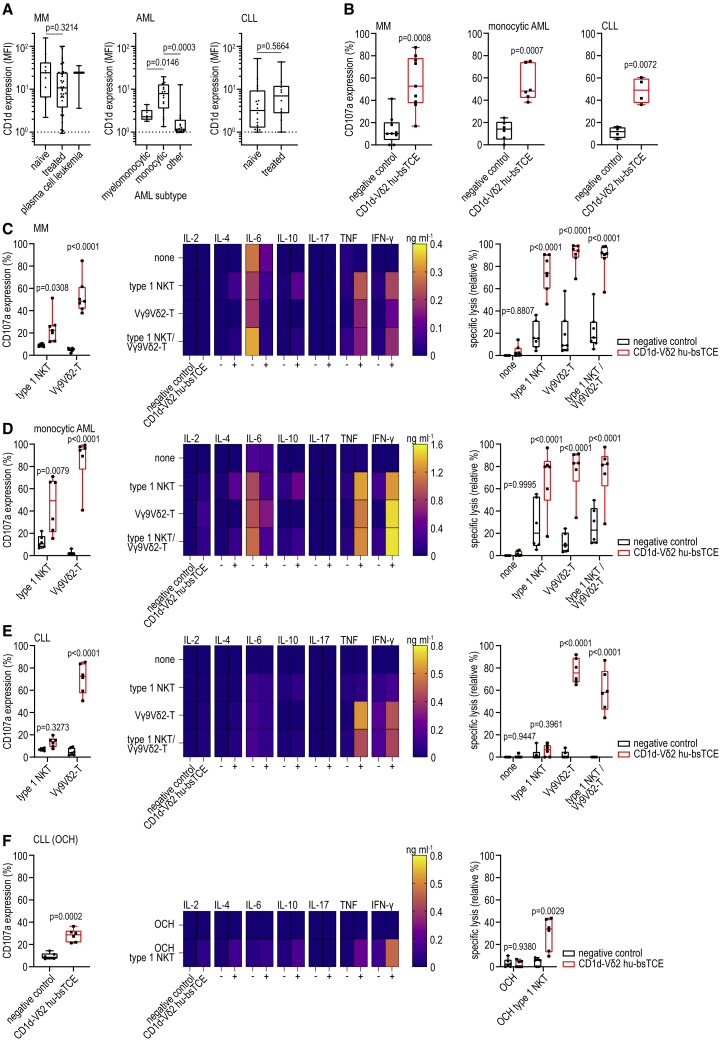
CD1d-Vδ2 bsTCE induces effector cell degranulation and cytotoxicity against patient-derived MM, monocytic AML, and CLL cells (A) CD1d expression on patient MM (n = 52), AML (n = 38), and CLL cells (n = 29) subdivided into treatment-naive and treated (MM and CLL) or subtype (AML). MFI, geometric mean fluorescence index. (B) CD107a expression on autologous Vγ9Vδ2-T cells after 16-h culture of patient MM BMMC (n = 9), monocytic AML BMMC (n = 6), or CLL PBMCs (n = 4) ± 50 nM CD1d-Vδ2 hu-bsTCE. (C–F) CD107a expression on allogeneic type 1 NKT, Vγ9Vδ2-T cells, or a 1:1 mixture thereof, secreted cytokines, and specific lysis of patient MM (C, n = 7), monocytic AML (D, n = 6), and CLL (E and F, n = 6) cells after a 16-h (co-)culture of patient BMMCs (C and D) or patient PBMCs (E and F) ± indicated effector cells (effector:BMMC/PBMC ratio 1:2) ± 50 nM CD1d-Vδ2 hu-bsTCE. (F) Patient CLL PBMCs were pre-incubated with the synthetic glycolipid OCH (100 ng mL^−1^) for 4 h. Negative control indicates medium control. Box-and-whisker plots indicate median, 25th–75th percentiles, and minimum-maximum. Heat maps indicate the mean. Unpaired two-tailed t test (A), paired two-tailed t test (B and F), one-way ANOVA with Tukey multiple comparisons test (A), two-way ANOVA with Šídák multiple comparisons test (C–F). See also Figure S4.

Next we assessed the antitumor effects of CD1d-Vδ2 hu-bsTCE using patient-derived tumor samples of (myelo)monocytic and B cell lineage origin, as these generally express CD1d.^19^^,^^21^^,^^22^^,^^30^^,^^31^ In bone marrow mononuclear cells (BMMCs) of patients with MM and AML and PBMCs of patients with CLL total T, type 1 NKT and Vγ9Vδ2-T cell frequencies and tumor cell CD1d expression were assessed. MM CD1d expression was especially high in treatment-naive patients, which is in agreement with a previous report.^30^ AML CD1d expression was most pronounced on monocytic and myelomonocytic phenotypic subtypes, while CD1d was expressed on CLL cells in the majority of patients and appeared slightly higher in treated patients (Figure 5A). Within the T cell compartment in BMMC/PBMC no statistically significant differences in type 1 NKT and Vγ9Vδ2-T cell frequencies were noted between MM, AML, and CLL, although the T cell frequency (of mononuclear cells) was significantly higher in BMMCs of MM patients compared with BMMCs of AML and PBMCs of CLL patients (Figure S4A). Upon 16-h culture of patient-derived MM, monocytic AML, and CLL tumor samples with CD1d-Vδ2 hu-bsTCE, clear degranulation of patient Vγ9Vδ2-T cells was observed (Figure 5B). Due to the (very) low type 1 NKT cell frequency in the tumor samples, degranulation of autologous type 1 NKT cells could not be reliably determined.

In co-cultures of BMMCs or PBMCs from patients with MM, monocytic AML, or CLL and expanded healthy donor-derived type 1 NKT, Vγ9Vδ2-T, or a mixture (1:1) thereof, CD1d-Vδ2 hu-bsTCE induced robust type 1 NKT and/or Vγ9Vδ2-T cell degranulation and cytokine production; for type 1 NKT cells, both T_h_1 (TNF, IFN-γ, some IL-2) and T_h_2 type cytokines (IL-4, IL-10); for Vγ9Vδ2-T cells predominantly T_h_1 type cytokines (TNF, IFN-γ, some IL-2) (Figures 5C–5E and S4B–S4D). Notably, levels of IL-6, a central driver of CRS symptoms secondary to chimeric-Ag receptor (CAR)-T or bsTCE treatment,^32^ were consistently reduced in conditions where type 1 NKT and/or Vγ9Vδ2-T cells were engaged by CD1d-Vδ2 hu-bsTCE. Neither stimulation of (autologous or allogeneic) type 1 NKT nor Vγ9Vδ2-T cells using the CD1d-Vδ2 hu-bsTCE resulted in IL-17 production (Figures 5C–5E). The CD1d-Vδ2 hu-bsTCE-induced activation of type 1 NKT and/or Vγ9Vδ2-T cells resulted in significant lysis of patient-derived MM, AML, and CLL cells (Figures 5C–5E). Although there was a trend between tumor CD1d expression and type 1 NKT and Vγ9Vδ2-T cell-mediated lysis, this did not reach statistical significance (Figures S4E–S4G). Interestingly, and in contrast to observations using MM and AML tumor samples, the CD1d-Vδ2 hu-bsTCE only triggered minimal type 1 NKT cell activation and cytolytic activity in co-cultures with CLL cells (Figure 5E). Since we previously showed that VHH1D12-induced type 1 NKT cell activation critically depended on the presence of (weakly) agonistic glycolipid Ag in CD1d,^23^ we hypothesized that CLL cells could differ from MM and AML cells in the amount and/or type of glycolipid Ag loaded in CD1d. ER stress triggers an unfolded protein response (UPR) that has been linked to enhanced endogenous agonistic lipid Ag presentation in CD1d.^33^^,^^34^ As activation of the UPR in CLL seems only partial under basal conditions,^35^ we explored whether induction of ER stress in CLL cells could enhance agonistic lipid Ag loading in CD1d and thereby promote CD1d-Vδ2 hu-bsTCE-mediated type 1 NKT cell reactivity. However, pre-incubation of CLL cells with thapsigargin, previously shown to induce ER stress and trigger type 1 NKT activation,^33^^,^^34^ did not enhance type 1 NKT cell degranulation or CLL lysis (Figure S4H), indicating that CLL cells might be impaired in their ability to load endogenous agonistic lipid Ag despite (induction of) ER stress. In support, pre-incubation of CLL cells with the low-affinity α-GalCer analog OCH, which loads at the cell surface and thereby bypasses the intracellular loading machinery,^36^ enabled the CD1d-Vδ2 hu-bsTCE to induce type 1 NKT cell degranulation, cytokine production, and cytotoxicity toward patient-derived CLL cells (Figure 5F).

Overall, these data demonstrate that CD1d is expressed by the majority of patient-derived MM, (myelo)monocytic AML, and CLL cells and that CD1d-Vδ2 hu-bsTCE strongly enhanced type 1 NKT and Vγ9Vδ2-T cell activation, resulting in robust cytotoxic responses toward these cells. In CLL, type 1 NKT (but not Vγ9Vδ2-T) reactivity depended on the presence of (exogenous) agonistic lipid Ags in CD1d.

### CD1d-Vδ2 bsTCE improves survival in *in vivo* AML, MM, and T-ALL xenograft mouse models

**Figure 6:**
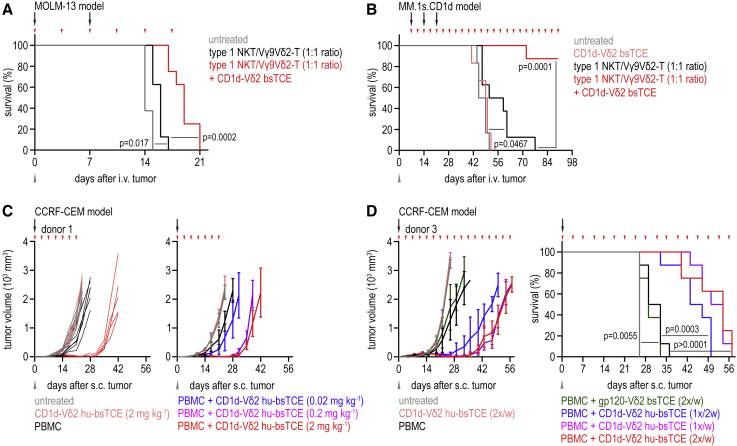
CD1d-Vδ2 bsTCE induces type 1 NKT and Vγ9Vδ2-T cell-mediated tumor protection *in vivo*, which improves survival (A) Survival curves of mice engrafted with MOLM-13 and infused with PBS (untreated) or a 1:1 mix of type 1 NKT/Vγ9Vδ2-T cells (10^7^ total cells, days 0 and 7) plus twice-weekly i.v. PBS or CD1d-Vδ2 bsTCE (5 mg kg^−1^) (n = 8). (B) Survival curves of mice engrafted with MM.1s.CD1d and infused with PBS (untreated, n = 6), or a 1:1 mix of type 1 NKT/Vγ9Vδ2-T cells (10^7^ total cells; days 7, 14, and 21; n = 8) plus twice-weekly i.p. PBS or CD1d-Vδ2 bsTCE (100 μg). (C) Tumor volume (mm^3^) in individual mice or group mean ± SD of s.c.-engrafted CCRF-CEM (10^7^ cells) mixed with PBS (untreated, n = 4) or PBMCs (5 × 10^6^ cells, n = 6) obtained from a healthy donor plus twice-weekly i.p. PBS or CD1d-Vδ2 bsTCE (indicated dose, up to day 21). (D) Mean ± SD tumor volume (mm^3^) and survival curves of mice s.c. engrafted with CCRF-CEM (10^7^ cells) mixed with PBS (untreated, n = 4) or PBMC (10^7^ cells, n = 8) obtained from a healthy donor plus i.p. PBS or indicated bsTCE (2 mg kg^−1^ at indicated interval). Gray arrowheads, tumor inoculation; black arrows, PBS or effector cell injection; red arrowheads, PBS or bsTCE infusion. Log rank test, two-tailed p values (A, B, and D). See also Figure S5.

To determine *in vivo* antitumor efficacy, we established disseminated AML and MM models via intravenous (i.v.) injection of MOLM-13 and MM.1s.CD1d in immunodeficient NOD scid gamma (NSG) mice. In the aggressively growing AML model, i.v. administration of mixed (1:1) human type 1 NKT/Vγ9Vδ2-T cells at day 0 and 7 had a modest effect on survival, which was significantly prolonged in combination with twice-weekly i.v. administration of CD1d-Vδ2 bsTCE (median survival of 14, 16, and 19 days, respectively) (Figure 6A). In the MM model, treatment was delayed until 7 days post tumor engraftment. Twice-weekly intraperitoneal (i.p.) administration of CD1d-Vδ2 bsTCE alone did not affect survival, whereas i.v. transfer of mixed (1:1) human type 1 NKT/Vγ9Vδ2-T cells (day 7, 14, and 21) significantly increased survival (median 47 and 55 days, respectively), nonetheless all mice succumbed to the disease (Figure 6B). A striking increase in survival was observed in mice receiving the combination of CD1d-Vδ2 bsTCE and type 1 NKT/Vγ9Vδ2-T cell transfer with 7 out of 8 mice surviving at the end of the study (Figure 6B). The one mouse that died likely succumbed to a small intra-peritoneal plasmacytoma causing intestinal obstruction. Persistence of type 1 NKT but not Vγ9Vδ2-T cells was observed in peripheral blood (Figures S5A and S5B). Persistence of peripheral blood Vγ9Vδ2-T cells appeared to be further reduced in mice treated with the CD1d-Vδ2 bsTCE, possibly as a result of redistribution to (tumor) tissue, TCR downregulation, or activation-induced cell death. Nonetheless, type 1 NKT but not Vγ9Vδ2-T cell persistence is in agreement with previous reports and relates to cross-reactivity of human type 1 NKT for mouse CD1d and absence of similar Vγ9Vδ2-T cell support due to lack of BTN2A1-BTN3A1 expression in mice.^8^^,^^37^^,^^38^

We next assessed the *in vivo* antitumor efficacy of the humanized CD1d-Vδ2 bsTCE using whole PBMCs admixed with CCRF-CEM cells (ratio 1:2; Vγ9Vδ2-T to T-ALL ratio 1:16 and 1:47 for donor 1 and 2 respectively) in a s.c. T-ALL model in NSG mice. In the absence of PBMCs, twice-weekly i.p. treatment with CD1d-Vδ2 hu-bsTCE (2 mg kg^−1^) did not inhibit tumor growth, whereas admixed PBMCs (in absence of bsTCE) slightly reduced tumor growth (Figures 6C and S5C). A remarkable, dose-dependent inhibition of tumor growth was observed in mice with admixed PBMCs treated with the CD1d-Vδ2 hu-bsTCE; tumor growth was only observed after discontinuation of bsTCE treatment (Figure 6C and S5C). We next evaluated the effect of different dosing intervals and continuation of dosing on tumor growth in this model (donor 3; PBMC to CCRF-CEM ratio 1:1; type 1 NKT and Vγ9Vδ2-T to T-ALL ratio 1:455 and 1:18, respectively), using two different doses of the CD1d-Vδ2 hu-bsTCE (2 mg kg^−1^ and 0.2 mg kg^−1^). Again, treatment with CD1d-Vδ2 hu-bsTCE (2 mg kg^−1^) alone had no effect, and PBMCs alone or combined with control gp120-Vδ2 bsTCE administration slightly reduced tumor growth, resulting in a minor increase in survival (median 26, 26, 31, and 29 days, respectively) (Figure 6D). Clear tumor growth inhibition and increased survival were observed in mice receiving the combination of PBMC and either of the CD1d-Vδ2 hu-bsTCE doses. Importantly, no significant differences in efficacy were observed between twice-weekly and weekly dosing, and, also, dosing once every 2 weeks resulted in a clear, albeit less pronounced, antitumor effect (median survival 54, 52, and 45 days, respectively, in mice receiving 2 mg kg^−1^, and median survival 48.5, 47, and 43 days, respectively, in mice receiving 0.2 mg kg^−1^) (Figures 6D and S5D).

Overall, these data show that CD1d-Vδ2 bsTCE improves survival in multiple *in vivo* hematologic malignancy models using either expanded type 1 NKT and Vγ9Vδ2-T cells or PBMCs as effector cells and demonstrate antitumor efficacy with intermittent dosing.

### Cross-reactive surrogate bsTCE in NHP induces Vγ9Vδ2-T cell activation and is well tolerated

**Figure 7:**
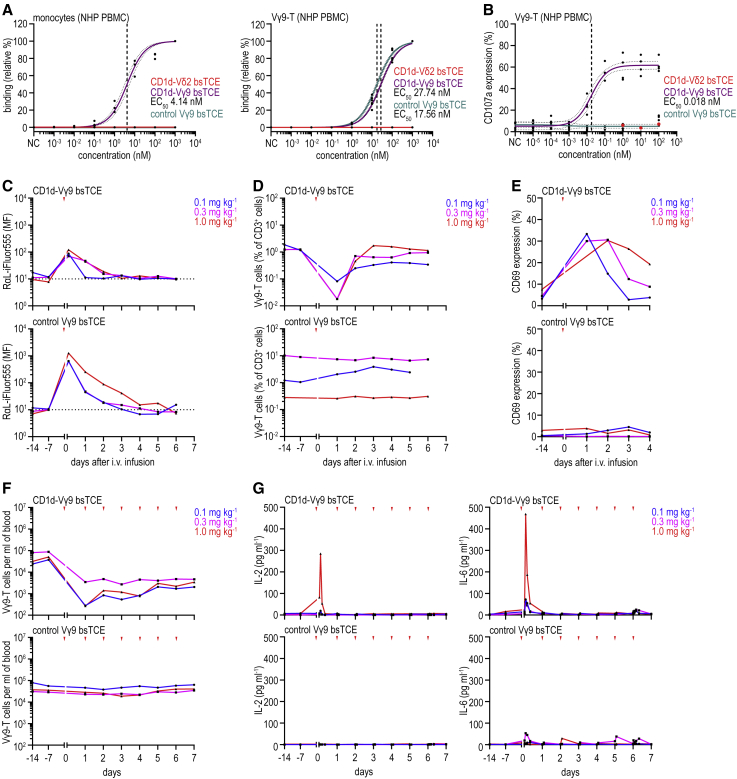
Infusion of a cross-reactive CD1d-Vγ9 bsTCE induces Vγ9-T cell activation and low levels of IL-6 release in NHPs (A) Relative binding of indicated biotinylated bsTCE (concentration range) to NHP monocytes and Vγ9-T cells (in PBMCs) detected by streptavidin-APC (n = 3). (B) CD107a expression on NHP Vγ9-T cells after 24-h culture of NHP PBMC ± concentration range of bsTCE (n = 3). (C) Binding of indicated bsTCE to NHP Vγ9-T cells over time (single-dose administration, 0.1, 0.3, or 1.0 mg kg^−1^ i.v.), detected by rabbit-anti-llama (RαL)-iFluor555. (D and E) NHP Vγ9-T cell percentage (of CD3^+^ cells) and expression of CD69 before and after a single i.v. dose (0.1, 0.3, or 1.0 mg kg^−1^) of indicated bsTCE. (F) NHP Vγ9-T cell frequency over time before i.v. dose (seven daily doses; 0.1, 0.3, or 1.0 mg kg^−1^ i.v.) of indicated bsTCE. (G) Pre- and post-dose plasma cytokine levels (0.5, 2, 4, 8, and 24 h after the first and last dose and 2 and 24 h after the remaining doses, 0.1, 0.3, or 1.0 mg kg^−1^) of indicated bsTCE. NC indicates PBS (A) or medium control (B). Red arrowheads, CD1d-Vγ9 bsTCE or control Vγ9 bsTCE infusion. For *in vivo*, n = 1 per concentration per bsTCE. MF, geometric mean fluorescence. Non-linear regression with 95% confidence bands (dotted lines) and EC_50s_ (dashed lines) (A and B). See also Figure S6 and S7.

To evaluate tolerability of CD1d-targeted Vγ9Vδ2-T cell engagement, we explored pharmacokinetic (PK) and pharmacodynamic (PD) parameters in NHP, which have a pAg-reactive Vγ9Vδ2-T cell population highly homologous to that of human.^39^^,^^40^ The binding arms of the CD1d-Vδ2 bsTCE (i.e., CD1d VHH1D12 and Vδ2-TCR VHH5C8) were not cross-reactive to *Macaca mulatta* and/or *Macaca fascicularis* (origin China, Mauritius, and Vietnam) CD1d or Vγ9Vδ2-T cells (Figure S6A). We therefore screened CD1d VHH1D22 (which binds a different epitope on CD1d and does not activate type 1 NKT) and a panel of Vγ9- and Vδ2-TCR-specific VHHs for NHP cross-reactivity.^23^^,^^41^^,^^42^ CD1d VHH1D22 was cross-reactive to NHP CD1d, whereas none of the Vγ9- and Vδ2-TCR-specific VHHs were cross-reactive with either *M. mulatta* or *M. fascicularis* Vγ9Vδ2-T cells (Figure S6A). Since no Vδ2-TCR mAbs with relevant cross-reactivity have been reported, we investigated anti-Vγ9-TCR mAb 7A5, for which reactivity with *M. mulatta* was demonstrated.^40^ The 7A5 single-chain variable fragment (scFv) was linked to the C terminus of CD1d VHH1D22. Binding properties of this CD1d-Vγ9 bsTCE and a control Vγ9 bsTCE (containing the non-cross-reactive CD1d VHH1D12) were tested. Indeed, only the CD1d-Vγ9 bsTCE specifically bound with low-nanomolar EC_50_ to CD1d^+^ NHP monocytes and nanomolar-range EC_50_ to NHP Vγ9Vδ2-T cells (Figures 7A and S6B). Binding EC_50_ of all three bsTCEs to human monocytes was equal, whereas binding EC_50_ of the Vγ9 bsTCEs to human Vγ9Vδ2-T cells was ∼30- to 60-fold higher compared with CD1d-Vδ2 bsTCE; however, this did not affect degranulation EC_50_ and tumor lysis (Figures 7A and S6C–S6E). The CD1d-Vγ9 bsTCE triggered NHP Vγ9Vδ2-T cell degranulation with an EC_50_ in the low-picomolar range (Figure 7B), demonstrating functional equivalence for triggering monkey and human Vγ9Vδ2-T cell activation.

Next, CD1d-Vγ9 bsTCE and the control Vγ9 bsTCE were tested in an NHP single and multiple dosing (0.1, 0.3, and 1.0 mg kg^−1^) study. As expected, and related to their small size and absence of an Fc domain, a single escalating dose of either bsTCE showed a short plasma half-life (3.1–12.8 h and 3.6–27.2 h, respectively), rapid plasma clearance, and large apparent volume of distribution (Figure S6F). Importantly, dose-dependent binding to peripheral blood Vγ9-T cells was observed up to several days after injection, and was particularly prominent for the control Vγ9 bsTCE, which was detectable on Vγ9-T cells for up to 5 days (Figure 7C). The shorter (detectable) duration and lower maximum binding of the CD1d-Vγ9 bsTCE likely reflects differences in the size of the Ag pool (CD1d and Vγ9-TCR versus Vγ9-TCR alone) and differences in Vγ9-T cell activation. Indeed, infusion of CD1d-Vγ9 bsTCE, but not the control bsTCE, induced a transient decrease in peripheral blood Vγ9-T cells with a concomitant upregulation of the activation marker CD69 (Figures 7D, 7E, and S6G).

Also, in the multiple-dosing study (seven daily doses), the binding intensity of the CD1d-Vγ9 bsTCE to peripheral blood Vγ9-T cells was lower compared with that observed with the control Vγ9 bsTCE, although binding remained detectable throughout the study at both the 0.3- and 1.0-mg kg^−1^ dose levels (Figure S7A). While the CD1d-Vγ9 bsTCE caused a decrease in peripheral blood and lymph node Vγ9-T cell numbers during the multi-dose study, no alterations in other PBMC subsets, including B cells, T cells, and monocytes, were observed (Figures 7F, S7B, and S7C). Importantly, infusion of CD1d-Vγ9 bsTCE (but not control Vγ9 bsTCE) induced a moderate dose-dependent increase in several proinflammatory cytokines (IL-2, TNF, IL-15), IL-6, and the chemokine CCL2, but only after the first administration (Figures 7G and S7D; Table S3). Body weights were not affected by the bsTCE and clinical observations and hematological and biochemical analyses did not show bsTCE-related toxicities (Table S4). Postmortem autopsy showed only local injection site inflammation for both constructs, which likely reflects repetitive infusion procedure-related injuries. No macroscopic findings were considered related to the bsTCE and no unscheduled deaths occurred during the study.

Overall, these data show that the fully NHP cross-reactive CD1d-Vγ9 bsTCE has a rapid plasma clearance but prolonged on-target binding, triggering clear Vγ9-T cell engagement, and was well tolerated in NHP.

## Discussion

CD1d is a (glyco)lipid Ag-presenting molecule frequently expressed by malignant cells of myelomonocytic and B cell lineage origin and some solid malignancies (e.g., renal cell carcinoma, medulloblastoma, and glioma), and may promote tumor growth via the presentation of (low-affinity) lipid Ags causing immunosuppressive cytokine release by type 1 NKT cells and engagement of protumoral diverse NKT cells.^10^^,^^19^^,^^21^^,^^22^^,^^30^^,^^31^ We demonstrate that a bsTCE specific for both CD1d and Vδ2 can induce strong CD1d-restricted activation of both Vγ9Vδ2-T and type 1 NKT cells, and simultaneously blocks the activation of CD1d-restricted diverse NKT cells. The CD1d-Vδ2 bsTCE triggered type 1 NKT and Vγ9Vδ2-T cells to secrete proinflammatory cytokines, with IFN-γ production being further boosted in the presence of both type 1 NKT and Vγ9Vδ2-T cells, possibly due to the reported stimulatory effects of type 1 NKT on Vγ9Vδ2-T cells.^43^ Such skewing toward T_h_1 cytokine responses is deemed important for downstream effector cell activation and has been linked to an improved response in several immunotherapy studies.^44^^,^^45^ The frequency of type 1 NKT and Vγ9Vδ2-T cells in patient tumor samples was within the range of those reported in healthy human donors and, importantly, Vγ9Vδ2-T cells exposed to CD1d-Vδ2 bsTCE in such samples could be readily activated. Of note, IL-17 production, associated with tumor-promoting activity,^46^ was not observed upon CD1d-Vδ2 bsTCE-mediated (autologous) type 1 NKT and Vγ9Vδ2-T cell activation. CD1d-Vδ2 bsTCE-induced type 1 NKT and Vγ9Vδ2-T cell activation resulted in robust target cell lysis at low E:T ratios, indicating serial killing, and was accompanied by expansion of both type 1 NKT and Vγ9Vδ2-T cells even in the absence of exogenous growth-promoting cytokines. This is relevant as effector cell expansion positively relates to therapy outcome in both CAR-T cell and bsTCE therapy in patients.^47^^,^^48^

Expression of CD1d on tumor cells was confirmed in the majority of MM, (myelo)monocytic AML, and CLL patients, and the CD1d-Vδ2 bsTCE-mediated engagement of type 1 NKT and Vγ9Vδ2-T cells resulting in a T_h_1 cytokine response and robust tumor cell lysis was also observed using patient-derived tumor samples. Of interest was the observation that the CD1d-Vδ2 bsTCE only triggered limited type 1 NKT cell activation in co-culture with patient-derived CLL cells. As CD1d expression in CLL appears to increase in more advanced stages of the disease,^19^ it is tempting to speculate that CLL cells exploit the use of a distinct lipid repertoire in CD1d to circumvent type 1 NKT cell recognition, potentially in favor of recognition by pro-tumor diverse NKT cell populations. Indeed, type 1 NKT reactivity to CD1d-Vδ2 bsTCE could be restored in co-cultures with CLL cells loaded with exogenous (low-affinity) lipid Ags.

Low-MW/low-affinity bsTCEs, such as blinatumomab, require continuous infusion to be effective.^49^ Despite the small size of the CD1d-Vδ2 bsTCE, which is below the renal threshold for first-pass clearance,^25^ antitumor activity was still evident using an administration frequency of only once every 2 weeks in a s.c. T-ALL/PBMC model. Combined with the low-nanomolar (type 1 NKT) to low-picomolar (Vγ9Vδ2-T) EC_50s_ observed in *in vitro* experiments, and prolonged binding of the cross-reactive bsTCE to peripheral blood Vγ9Vδ2-T cells despite a short plasma half-life as observed in the NHP study, these data indicate an extended functional half-life and are supportive of an intermittent-dosing approach of the CD1d-Vδ2 bsTCE in humans.

Common adverse events of bsTCE and CAR-T cell therapies include CRS and ICANS that are potentially life threatening and frequently mandate dose adjustment and/or immunosuppressive measures.^1^^,^^3^ Both systemic activation (using glycolipid Ag- and pAg-based approaches) and adoptive transfer of type 1 NKT and Vγ9Vδ2-T cells were found to have a good safety profile in earlier clinical studies,^11^^,^^15^^,^^22^ suggesting a low risk for CRS and/or ICANS upon CD1d-Vδ2 bsTCE infusion. Nonetheless, we assessed tolerability of CD1d-γδ T cell engagement in an NHP preclinical safety study. Because CD1d VHH1D22 does not share the type 1 NKT cell-stimulatory property of CD1d VHH1D12,^23^ this aspect could not be studied. However, Vγ9Vδ2-T cells represent the substantially larger effector cell population of the two; therefore, we believe it is likely that their engagement is the dominant factor to consider. Infusion of both single and multiple doses of a cross-reactive CD1d-Vγ9 bsTCE, resulting in clear Vγ9Vδ2-T cell engagement, was well tolerated in NHPs even at high doses of 1 mg kg^−1^. Importantly, blood plasma analysis showed low levels of IL-6 release only after the first and highest dose, indicating a low risk for CRS. Furthermore, no depletion of CD1d^+^ B cells and monocytes was observed. This is in agreement with our *in vitro* results showing preferential lysis of malignant cells while B cells and monocytes were relatively spared, implicating a low risk for on-target off-tumor toxicity.

Collectively, we have generated a first-in-class, off-the-shelf, humanized CD1d-Vδ2 bsTCE that selectively engages both type 1 NKT and Vγ9Vδ2-T cells to trigger a potent antitumor response to CD1d-expressing tumor cells. Considering the expression of CD1d in CLL, MM, and AML, and the favorable tolerability profile of the surrogate engager in NHPs, the CD1d-Vδ2 hu-bsTCE was termed LAVA-051 and selected for evaluation in a first-in-human clinical phase 1/2a study in patients with CLL, MM, or AML that are refractory to prior therapy (ClinicalTrials.gov identifier: NCT04887259).

### Limitations of the study

Our study has several limitations. First, as mice do not have a pAg-responsive Vγ9Vδ2-T cell population^8^ and the CD1d-specific VHH1D12 triggers activation of human but not mouse type 1 NKT cells,^23^ xenograft (immunodeficient) mouse models had to be used in which downstream immune activation cannot be fully assessed, limiting their translational relevance. Second, since the CD1d-Vδ2 bsTCE lacked NHP cross-reactivity, a surrogate CD1d-Vγ9 bsTCE was used to explore tolerability in NHP. As the CD1d-binding arm lacked the ability to stimulate type 1 NKT cells, the impact of type 1 NKT cell activation on tolerability could not be explored. Third, while our study focused on assessing the anticancer potential of a bsTCE that selectively engages type 1 NKT and Vγ9Vδ2-T cells toward CD1d-expressing hematological malignances, it did not directly compare *in vitro* and *in vivo* efficacy or tolerability in NHPs with a CD3-targeting bsTCE directed against CD1d.

## STAR★Methods

### Key resources table

**Table:** 

REAGENT or RESOURCE	SOURCE	IDENTIFIER
**Antibodies**		

Mouse anti-human CD1d-unconjugated (clone 51.1)	Biolegend	Cat# 350321; RRID:
Mouse anti-human CD1d- APC (clone 51.1)	Biolegend	Cat# 350308; RRID:AB_10642829
Mouse anti-human CD1d-PE (clone 51.1),	Thermo Fisher Scientific	Cat# 12-0016-42; RRID:
Mouse anti-human CD3-FITC (clone SK7)	BD Biosciences	Cat# 345763; RRID:AB_2811220
Mouse anti-human CD3^−^ APC (clone SK7)	BD Biosciences	Cat# 345767; RRID:AB_2833003
Mouse anti-human CD3-APC-H7 (clone SK7)	BD Biosciences	Cat# 560176; RRID:AB_1645475
Mouse anti-human CD3-BV421 (clone SK7)	BD Biosciences	Cat# 563797; RRID:AB_2744383
Mouse anti-human CD3-AF700 (clone SP34-2)	BD Biosciences	Cat# 557917; RRID:AB_396938
Mouse anti-human CD3-BUV395 (clone SP34-2)	BD Biosciences	Cat# 564117; RRID:AB_2738603
Mouse anti-human CD3-BV711 (clone UCHT1)	BD Biosciences	Cat# 563724; RRID:AB_2744392
Mouse anti-human CD5-BV605 (clone UCHT2)	BD Biosciences	Cat# 563945; RRID:AB_2738500
Mouse anti-human CD5-BV605 (clone L17F12)	Biolegend	Cat# 364020; RRID:AB_2565941
Mouse anti-human CD7-APC (clone M-T701)	BD Bioscience	Cat# 653311; RRID:AB_2870351
Mouse anti-human CD11b-PE-Cy7 (clone ICRF44)	BD Bioscience	Cat# 557743; RRID:AB_396849
Mouse anti-human CD13-BV421 (clone WM15)	BD biosciences	Cat# 562596; RRID:AB_2737672
Mouse anti-human CD14-FITC (clone MΦP9)	BD Biosciences	Cat# 345784; RRID:AB_2868810
Mouse anti-human CD14-PE-Dazzle594 (clone M5E2)	Biolegend	Cat# 301852; RRID:AB_2629576
Mouse anti-human CD14-PE-Cy7 (clone 63D3(	Biolegend	Cat# 367112; RRID:AB_2566714
Mouse anti-human CD19-PC7 (clone J3-119)	Beckman Coulter	Cat# IM3628
Mouse anti-human CD19-PE-CF594 (clone HIB19)	BD Biosciences	Cat# 562294; RRID:AB_11154408
Mouse anti-human CD19-APC (clone SJ25C1)	BD biosciences	Cat# 345791; RRID:AB_2868817
Mouse anti-human CD19-BV605 (clone SJ25C1)	BD biosciences	Cat# 740394; RRID:AB_2740124
Mouse anti-human CD20-FITC (clone 2H7)	BD Biosciences	Cat# 555622; RRID:AB_395988
Mouse anti-human CD20-AF700 (clone 2H7)	Biolegend	Cat# 303222
Mouse anti-human CD33-PE-CF594 (clone WM53)	BD Biosciences	Cat# 562492; RRID:AB_2713912
Mouse anti-human CD33-BV421 (clone WM53)	BD Biosciences	Cat# 562854; RRID:AB_2737405
Mouse anti-human CD34 PerCPCy5.5 (clone 8G12)	BD Biosciences	Cat# 347222; RRID:AB_2868843
Mouse anti-human CD34-APC (clone 581)	BD Biosciences	Cat# 555824; RRID:AB_398614
Mouse anti-human CD38-FITC (clone HB7)	BD Biosciences	Cat# 340909; RRID:AB_2868744
Mouse anti-human CD38-APC (clone HB7)	BD Biosciences	Cat# 340439; RRID:AB_400512
Mouse anti-human CD45-AF700 (clone HI30)	Biolegend	Cat# 304024; RRID:AB_493761
Mouse anti-human CD45-KO (clone J.33)	Beckman Coulter	Cat# B36294; RRID:AB_2833027
Mouse anti-human CD56-PC7 (clone N901)	Beckman Coulter	Cat# A21692; RRID:AB_2892144
Mouse anti-human CD56-APC (clone HCD56)	Biolegend	Cat# 318310; RRID:AB_604106
Mouse anti-human CD56-BV510 (clone NCAM16.2)	BD biosciences	Cat# 563041; RRID:AB_2732786
Mouse anti-human CD69-PE (clone FN50)	BD biosciences	Cat# 555531; RRID:AB_395916
Mouse anti-human CD69-APC-Fire750 (clone FN50)	Biolegend	Cat# 310946; RRID:AB_2616709
Mouse anti-human CD81 APC-H7 (clone JS-81)	BD Bioscience	Cat# 656647; RRID:AB_2870414
Mouse anti-human CD107a-PE (clone H4A3)	Miltenyi Biotec	Cat# 130-095-515; RRID:AB_10828805
Mouse anti-human CD107a-PE (clone H4A3)	Thermo Fischer Scientific	Cat# 12-1079-42; RRID:AB_10853326
Mouse anti-human CD117-PE-Cy7 (clone 104D2)	BD Biosciences	Cat# 339217; RRID:AB_2868720
Mouse anti-human CD117-APC (clone 104D2)	BD Biosciences	Cat# 333233; RRID:AB_2868677
Mouse anti-human CD117-BV650 (clone 104D2)	BD Biosciences	Cat# 563859; RRID:AB_2738453
Mouse anti-human CD138-FITC (clone MI15)	Biolegend	Cat# 356507; RRID:AB_2561881
Mouse anti-human CD138-BV421 (clone MI15)	BD biosciences	Cat# 562935; RRID:AB_2737904
Mouse anti-human TRAV10-PC7 (clone C15)	Beckman Coulter	Cat# A66907
Mouse anti-human TRBV25-1-PE (clone C21)	Beckman Coulter	Cat# IM2290; RRID:AB_131325
Mouse anti-human TRDV2-FITC (clone Immu389)	Beckman Coulter	Cat# IM1464; RRID:AB_131019
Mouse anti-human TRDV2-BV711 (clone B6)	Biolegend	Cat# 331412; RRID:AB_2565421
Mouse anti-human TRGV9-FITC (clone B3)	BD Biosciences	Cat# 555732; RRID:AB_396075
Mouse anti-human TRGV9-FITC (clone Immu360)	Beckman Coulter	Cat# IM1463; RRID:AB_130871
Mouse anti-human TRGV9-PE (clone B3)	Biolegend	Cat# 331308; RRID:AB_1236408
Mouse anti-human TRGV9-APC (clone B3)	Biolegend	Cat# 331310; RRID:AB_2057504
Mouse anti-human pan-γδTCR-BV421 (clone B1)	BD Bioscience	Cat# 562560; RRID:AB_2737655
Mouse anti-human HLA-DR-V450 (clone L243)	BioLegend	Cat# 655874; RRID:AB_2716783
Mouse anti-human HLA-DR-BV786 (clone L243)	BioLegend	Cat# 307642; RRID:AB_2563461
Goat anti-Llama IgG-heavy and light chain Antibody (GαL)-FITC (polyclonal)	Bethyl laboratories	Cat# A160-100F; RRID:AB_67106
Rabbit anti-Llama VHH (RαL)-iFluor488 (cocktail)	Genscript	Cat# A02021
Rabbit anti-Llama VHH (RαL)-iFluor555 mAb (clone 96A3F5)	Genscript	Cat# A01994
Rabbit anti-Llama VHH (RαL)-iFluor555 mAb (clone 96A3F5)	Genscript	Cat# A01863
Rat anti-mouse CD45-FITC (clone 30F11)	BD Biosciences	Cat# 553079; RRID:AB_394609
Rat anti-Mouse CD16/CD32-unconjugated (clone 2.4G2)	BD Biosciences	Cat# 553141; RRID:AB_394656
human FcR blocking reagent	Miltenyi Biotec	Cat# 130-059-901; RRID:AB_2892112

**Biological samples**		

healthy human donor PBMC	Sanquin blood supply, the Netherlands	N/A
healthy human donor PBMC	AllCells, China	N/A
healthy human donor PBMC	Milestone Shanghai Biological Science & Technology, China	N/A
MM patient BMMC	Amsterdam UMC, the Netherlands	N/A
MM patient BMMC	Cureline tissue bank, San Francisco, USA	N/A
AML patient BMMC	Amsterdam UMC	N/A
AML patient BMMC	Centre de Ressources Biologiques des Hospices Civils, Lyon, France	N/A
CLL patient PBMC	Amsterdam UMC	N/A
*M. fascicularis* (origin China, Mauritius, and Vietnam) PBMC	CITox, Miserey, France	N/A
*M. mulatta* PBMC	CITox, Miserey, France	N/A

**Chemicals, peptides, and recombinant proteins**		

α-GalCer	Avanti Polar Lipids	Cat# 867000P
OCH	AdipoGen	Cat # AG-CR1-3593
Sulfatide C24	Avanti Polar Lipids	Cat# 860871
CD1d(α-GalCer)-PE tetramer	Lameris et al., 2020^23^	N/A
CD1d(α-GalCer)-APC tetramer	Lameris et al., 2020^23^	N/A
CD1d(α-GalCer)-BV421 tetramer	Lameris et al., 2020^23^	N/A
CD1d(α-GalCer)-BV711 tetramer	Lameris et al., 2020^23^	N/A
CD1d(endogenous)-PE tetramer	Lameris et al., 2020^23^	N/A
CD1d(sulfatide)-PE tetramer	Lameris et al., 2020^23^	N/A
Thapsigargin	Sigma Aldrich	Cat# T9033
streptavidin-APC	Thermo Fischer Scientific	Cat# 17-4317-82
streptavidin-PE	Thermo Fischer Scientific	Cat# S866
NHS-D-biotin	Sigma Aldrich	Cat# H1759
7-amino-actinomycin D (7-AAD)	Sigma	Cat# A9400
Annexin V-FITC	VPS diagnostics	Cat# A700
pacific blue succinimidyl ester (PBSE)	Thermo Fisher Scientific	Cat# P10163
RPMI-1640	Gibco, Thermo Fisher Scientific	Cat# 22400089
IMDM	Gibco, Thermo Fisher Scientific	Cat# 12440061
DMEM	Gibco, Thermo Fisher Scientific	Cat# 41965039
FCS	Biological Industries	Cat# 04-007-1A
Human AB serum	MP biomedicals	Cat# 092930949
β-mercaptoethanol	Merck	Cat# 805740
sodium penicillin, streptomycin sulphate and L-glutamine (100×)	Gibco, Thermo Fisher Scientific	Cat# 10378016
Recombinant human IL-2	Novartis	N/A
Recombinant human IL-4	R&D Systems	Cat# 204-IL/CF
Recombinant human IL-7	R&D Systems	Cat# 207-IL-025
Recombinant human IL-15	eBioscience	Cat# 34-8159-85
GM-CSF	Sanofi Leukine	N/A
LPS	Sigma	Cat# L6529
phytohaemagglutinin (PHA)	Thermo Fisher Scientific	Cat# R30852801
Anti-CD1d VHH (clone VHH1D12)	Lameris et al., 2020^23^	N/A
Anti-CD1d VHH (clone VHH1D22)	Lameris et al., 2016^41^	N/A
Anti-Vγ9 and/or Vδ2 TCR VHH (panel)	de Bruin et al., 2016^42^	N/A
CD1d-Vδ2 bsVHH (clone VHH1D12-VHH5C8)	This paper	N/A
Anti-CD1d VHH WT and variant 1–10 and Q1E (clone VHH1D12)	This paper	N/A
Anti-Vδ2-TCR VHH WT and variant 1–10 (clone VHH5C8)	This paper	N/A
CD1d-Vδ2 hu-bsVHH (LAVA-051)	This paper	N/A
Control gp120-Vδ2 bsVHH (clone VHHL8CJ3-VHH5C8)	This paper	N/A
CD1d-Vγ9 bsTCE (VHH1D22-scFv7A5)	This paper	N/A
Control Vγ9 bsTCE (VHH1D12-scFv7A5)	This paper	N/A
Lymphoprep	Stemcell tenchnologies	Cat# 07861

**Critical commercial assays**		

Counting beads	Thermo Fisher Scientific	Cat# 01-1234-42
BA Human Th1/Th2/Th17 Kit	BD Biosciences	Cat# 560484; RRID:AB_2869353
Annexin V Binding Buffer, 10X concentrate	BD Biosciences	Cat# 556454; RRID:AB_2869074
10X RBC Lysis Buffer	Thermo Fisher Scientific	Cat# 00-4300-54
anti-mous IgG microBeads	Miltenyi	Cat # 130-048-401; RRID:AB_244361
Anti-type 1 NKT microbeads	Miltenyi	Cat # 130-094-842
Anti-PE microbeads	Miltenyi	Cat # 130-048-801; RRID:AB_244373
Pan monocyte isolation kit	Miltenyi	Cat # 130-096-537
Human gamma/delta T cell isolation kit	Stemcell tech	Cat # 19255

**Experimental models: Cell lines**		

Human: CCRF-CEM	ATCC	Cat# CRM-CCL-119; RRID:CVCL_0207
Human: MOLM-13	DSMZ	Cat# ACC-554; RRID:CVCL_2119
Human: NOMO-1	DSMZ	Cat# ACC-542; RRID:CVCL_1609
Human: THP-1	Sigma	Cat# 88081201; RRID:CVCL_0006
Human: MM.1s whether or not expressing mCherry/Luc and/or CD1d)	Lameris et al., 2020^23^	N/A
Human: J.RT3-T3.5 (JRT3) expressing CD1d(sulfatide)-restricted TCR DP10.7	Luoma et al., 2013^50^	N/A
Human: HeLa	M. Kronenberg	N/A
Human: HeLa.CD1d	M. Kronenberg	N/A
Human: A-431	ATCC	Cat# CRL-1555; RRID:CVCL_0037
Human: JY cells	N/A	RRID:CVCL_0108

**Experimental models: Organisms/strains**		

Mouse: NOD *scid* gamma (AML and MM model)	Charles River	Cat# JAX:005557; RRID:IMSR_JAX:005557
Mouse: NOD *scid* gamma (T-ALL model)	Vital River laboratories	Cat# JAX:005557; RRID:IMSR_JAX:005557
NHP: *M. fascicularis*	CrownBio	N/A

**Software and algorithms**		

Kaluza v1.3	Beckman Coulter	https://www.beckman.com/flow-cytometry/software/kaluza; RRID:SCR_016182
FlowJo v10	BD Biosciences	https://www.flowjo.com/; RRID:SCR_008520
FCAP array v3.0	BD Biosciences	https://www.bdbiosciences.com/en-us/products/instruments/software-informatics/instrument-software/fcap-array-software-v3-0.652099w
GraphPad Prism v9.1.0	GraphPad	https://www.graphpad.com/; RRID:SCR_002798
PK Solver software version 2.0	Zhang et al., 2010^51^	N/A

### Resource availability

#### Lead contact

Further information and request for resources and reagents should be directed to and will be fulfilled by Hans J. van der Vliet: h.vandervliet@lavatherapeutics.com.

#### Materials availability

Antibodies generated for this study are available upon request and completion of a material transfer agreement.

### Experimental model and subject details

#### Cell lines

The T-ALL cell line CCRF-CEM was obtained from the ATCC and grown in RPMI-1640 supplemented with 10% (v/v) fetal calf serum (FCS), 0.05 mM β-mercaptoethanol (β-ME), and 100 IU mL^−1^ sodium penicillin, 100 μg mL^−1^ streptomycin sulphate and 2.0 mM L-glutamine. The AML cell lines MOLM-13 and NOMO-1 were obtained from the DSMZ and THP-1 from Sigma, and grown in RPMI-1640 supplemented as above. The MM cell line MM.1s, wild type (WT) or transduced with the lentiviral plasmid FUW-mCherry/luc^52^^,^^53^ and electroporated with full length single chain β2m-CD1d pcDNA3.1 vector (referred to as MM.1s.WT and MM.1s.CD1d)^23^^,^^54^ was grown in RPMI-1640 supplemented as above. The lymphoblastic cell line C1R, WT or stably transduced with CD1d,^55^ was grown in IMDM supplemented as above. The Epstein-Barr Virus transformed lymphoblastoid cell line JY was grown in IMDM supplemented as above. The βTCR-deficient cell line J.RT3-T3.5 (JRT3) expressing CD1d(sulfatide)-restricted TCR DP10.7^54^ was grown in RPMI-1640 supplemented as above. The epithelial cell line HeLa (WT and transfected with CD1d) was a gift from dr. M. Kronenberg and grown in DMEM supplemented as above. The epithelial cell line A-431 was obtained from the ATCC and grown in DMEM supplemented as above. Cell lines were confirmed to be Mycoplasma-free. Authentication of CCRF-CEM and MM.1s transfectants (BaseClear and Eurofins) showed no signs of contamination or misidentification.

#### Human samples

PBMC from healthy donors were isolated from venous blood by density gradient centrifugation, obtained from Sanquin blood supply under written informed consent (the Netherlands) or commercially acquired (T-ALL mouse model only; AllCells, China or Milestone Shanghai Biological Science & Technology, China). Bone marrow mononuclear cells (BMMC) from patients with MM (n = 52, mean age 62 years, female/male 47/53%, treatment naive 27% (from 3 patients no age and from 1 patient no gender was available)) or patients with AML (n = 59, mean age 57 years, female/male 37/63%, treatment naive 92%) were isolated from bone marrow samples by density gradient centrifugation, obtained after approval by the institutional review board (medical ethical committee Amsterdam UMC, location VUmc) and written informed consent from the patients or commercially acquired (MM samples from Cureline tissue bank, San Francisco, USA; AML samples from Centre de Resources Biologiques des Hospices Civils, Lyon, France). PBMC from CLL patients (n = 59, mean age 66 years, female/male 29/71%, treatment naïve 76% (from 1 patient no age and gender was available)) were isolated from venous blood by density gradient centrifugation, obtained after approval by the institutional review board (medical ethical committee Amsterdam UMC, location AMC) and written informed consent from the patients.

#### Mouse models

Disseminated AML and MM models were established by i.v. transfer of AML and CD1d^+^ MM cells, respectively, into NOD *scid* gamma (NSG) mice (Charles River). For the AML model, female 8-9-week-old NSG mice were i.v. injected with 0.25×10^6^ MOLM-13 cells via the tail vein (day 0). One hour later and on day 7 PBS or a mixture of 0.5×10^7^ human type 1 NKT and 0.5×10^7^ human Vγ9Vδ2-T cells were i.v. injected. Mice were twice weekly i.v. injected with CD1d-Vδ2 bsTCE (first dose 1h after effector cell infusion, 5 mg kg^−1^). Mice were euthanized when pre-set human endpoints were reached. All mice were maintained in a specific pathogen-free facility (12h light/12h dark cycle) and supplied with autoclaved bedding and *ad libitum* water and food. The AML *in vivo* study was performed by Oncodesign, France, and approved by the animal Care and Use Committee of Oncodesign (Oncomet) agreed by French authorities (CNREEA agreement number 91).

For the MM model, female 16-26-week-old NSG mice (Charles River) were irradiated with 2 Gy 24h prior to i.v. injection of 2.5 × 10^6^ MM.1s.CD1d cells via the tail vein (day 0). On day 7, 14, and 21 PBS or a mixture of 0.5×10^7^ human type 1 NKT and 0.5 × 10^7^ Vγ9Vδ2-T cells with or without CD1d-Vδ2 bsTCE (100 μg per mouse) were i.v. injected. Mice were twice weekly i.p. injected with PBS or CD1d-Vδ2 bsTCE (100 μg per mouse). Peripheral blood type 1 NKT (mCD45^−^hCD45^+^CD3^+^TRAV-10^+^) and Vγ9Vδ2-T cell frequencies (mCD45^−^hCD45^+^CD3^+^TRGV9^+^(TRDV2^+^)) were determined over time by flow cytometric bead-based cell counting. Mice were euthanized when pre-set human endpoints were reached. Blood in the one mouse, treated with type 1 NKT and Vγ9Vδ2-T cells plus CD1d-Vδ2 bsTCE, that was found dead could not be reliably analyzed due to clotting and was excluded from the analyses. The study was terminated 90 days post tumor engraftment. All mice were maintained as described above. Animal experiments were carried out in compliance with Dutch/European ethical guidelines and approved by the Dutch Central Authority for Scientific Procedures on Animals (permission number AVD114002016402).

A s.c. T-ALL model was established via s.c. inoculation of 1 × 10^7^ CCRF-CEM cells alone or mixed with 0.5–1.0×10^7^ (as indicated) healthy donor PBMC (day 0) in female 5-9-week old NSG mice (Vital River laboratories). Mice were i.p. injected with PBS or CD1d-Vδ2 hu-bsTCE (indicated dose and frequency). Tumor volumes were measured twice a week in two dimensions using a caliper, and the tumor volume (V) calculated using the formula: V = (W^2^×L)/2, where L is tumor length (the longest tumor dimension) and W is tumor width (the longest tumor dimension perpendicular to L). In the dose escalating part, mice were euthanized when the tumor volume of individual mice reached >3000 mm^3^, mean tumor volume of the groups reached >2000 mm^3^ or when pre-set human endpoints were reached. In the dosing interval study, mice were euthanized when the tumor volume of individual mice reached >2000 mm^3^ or when pre-set human endpoints were reached. All mice were maintained as described above. The T-ALL *in vivo* study was performed by Crown Bioscience, China, in compliance with the regulations of the Association for Assessment and Accreditation of Laboratory Animal Care (AAALAC) and approved by the Institutional Animal Care and Use Committee (IACUC) of Crown Bioscience.

#### NHP single and multiple dosing study

PK and PD of CD1d-Vγ9 bsTCE or control Vγ9-bsTCE were studied in 12 female *M. fascicularis*, at Crown Bioscience, Beijing, China. The study was divided in two phases. Phase 1: NHP received a single dose CD1d-Vγ9 bsTCE or control Vγ9 bsTCE (0.1 mg kg^−1^, 0.3 mg kg^−1^ or 1 mg kg^−1^ via a 30-min i.v. infusion, n = 1 animal per group) to determine PK values. Venous blood samples were collected pre-dose (day −14 and −7), 0, 0.5, 1, 2, 4, 6, and 8h, and day 1, 2, 3, 4, 5 and 6 post-infusion to determine PK values and/or flow cytometry analyses. Free plasma concentrations of the bsTCEs, measured using a free drug assay (Simoa, Quanterix) by ABL immunology (Lyon, France), were used to estimate PK parameters (PK Solver software version 2.0^55^). Peripheral blood Vγ9-T cell percentages (CD3^+^TRGV9^+^(RαL^+^)), CD69 expression, and binding of CD1d-Vγ9 bsTCE or control Vγ9 bsTCE to Vγ9Vδ2-T cells were determined over time by flow cytometry. Binding to CD1d^+^ cells could not reliably be detected since the used RαL antibody could not bind VHH1D22 when bound to CD1d.

Phase 2 (conducted in sequel of phase 1): NHP received 7 daily i.v. doses of CD1d-Vγ9 bsTCE or control Vγ9 bsTCE (0.1 mg kg^−1^, 0.3 mg kg^−1^ or 1 mg kg^−1^ via a 30-min i.v. infusion, n = 1 animal per group). Venous blood samples were collected pre-dose (day −14 and −7), 0.5, 2, 4, 8 and 24h after the first and last dose and 2 and 24h after the remaining doses. Hematological parameters (complete blood cell count), coagulation (prothrombin time, activated partial thromboplastin time, fibrinogen and thrombin time), chemistry (alanine aminotransferase, aspartate aminotransferase, total bilirubin, direct bilirubin, total protein, prealbumin, albumin, globulin, albumin/globulin ratio, alkaline phosphatase, gamma glutamyl transferase, adenosine deaminase, lactate dehydrogenase, blood urea nitrogen, serum creatinine, blood uric acid, cystatin) and cytokines (U-PLEX Biomarker Group 1 (NHP) Assays; IL-1β, IL-2, IL-4, IL-5, IL-8, IL-10, IL-12p70, TNF, IFN-γ and CCL2) were determined over time. Axillary lymph nodes were surgically collected under general anesthesia on day −14 and 24h after the final i.v. dose and processed to single cell suspensions. 24h after the 7^th^ infusion the animals were euthanized for autopsy. Peripheral blood and lymph node Vγ9-T cell frequencies and binding of CD1d-Vγ9 bsTCE or control Vγ9 bsTCE (CD3^+^TRGV9^+^(RαL^+^)), and T cell (CD3^+^), B cell (CD3^−^CD20^+^) and monocyte (CD3^−^CD14^+^) frequencies were determined over time by flow cytometry. Absolute numbers were calculated as follows: [target population ml^−1^ blood] = ([counts in target population gate (FACS)]/[Counts in leukocyte gate (FACS)]) x [white blood cell count ml^−1^].

NHP were maintained at 20–23°C, 40–70% humidity with a 12h light/12h dark cycle. All animals had free access to water and were fed twice daily with a nutritionally balanced diet supplemented with seasonal fruits. Animals were observed twice daily for clinical abnormalities. Body weight was recorded weekly. The study was approved by Crown Bioscience animal care and use committee (IACUC) and all procedures related to housing, handling, caring and treatment of the animals were performed in compliance with the guidelines approved by the AAALAC.

### Method details

#### Flow cytometry

All antibodies were used at empirically determined dilution factors. Staining was performed at 4°C in PBS supplemented with 0.1% BSA and 0.02% sodium azide unless otherwise stated. Analyses and cell sorting were performed using a FACS Canto II or FACS LSRFortessa (BD Biosciences). Data analysis was performed using Kaluza (Beckman Coulter), FCAP array and FlowJo (BD Biosciences).

#### Generation of bsTCE

Human CD1d-specific and human Vγ9-and Vδ2-TCR-specific VHH were generated and screened as previously described.^41^^,^^42^ To generate the CD1d-Vδ2 TCR bispecific VHH, CD1d (clone VHH1D12) and Vδ2-TCR (clone VHH5C8) specific VHHs were linked by a Gly_4_Ser(G_4_S)-linker (VHH1D12-VHH5C8 (further referred to as CD1d-Vδ2 bsTCE)) and purified, tagless, and endotoxin free CD1d-Vδ2 bsTCE was produced by UPE, the Netherlands. N-terminal positioning of VHH1D12 had a minimal functional advantage compared to C-terminal positioning in triggering type 1 NKT cell degranulation and was equally effective in triggering Vγ9Vδ2-T cell degranulation (data not shown). Control gp120-Vδ2 bsTCE (anti-gp120 VHHL8CJ3 which binds to glycoprotein (gp)120 of HIV-1)^56^ was linked by a G_4_S-linker to the N-terminus of anti-Vδ2 TCR VHH (clone VHH5C8)) and produced by UPE.

#### Binding assay

For *in vitro* binding experiments of monovalent VHH or bsTCE, ∼1×10^5^ cells (Vγ9Vδ2-T, αβ-T, MM.1s WT or MM.1s.CD1d cells were incubated with PBS (negative control) or indicated concentrations of the constructs for 30–45 min at 4°C, followed by extensive washing and incubation for 30 min at 4°C with GαL-FITC, RαL-VHH iFluor488 or RαL-VHH iFluor647, washed and analyzed by flow cytometry. The relative percentage of binding was calculated by dividing delta median fluorescence (MF) [condition] (MF condition minus MF 0 nM) by delta MF [1000 nM] (MF 1000 nM minus MF 0 nM) multiplied by 100. In case delta MF 0–1000 nM was ∼0, binding was set to 0% for all conditions.

#### Primary type 1 NKT and Vγ9Vδ2-T cell lines

Primary human type 1 NKT cells were generated as described previously.^57^ In brief, monocytes were isolated from PBMC of healthy donors by magnetic bead sorting using anti-CD14 microbeads and cultured in RPMI-1640 medium supplemented with 10% (v/v) FCS, 0.05 β-ME, and 100 IU mL^−1^ sodium penicillin, 100 μg mL^−1^ streptomycin sulfate, 2.0 mM L-glutamine, 1000 U ml^−1^ GM-CSF and 20 ng mL^−1^ rhIL-4 for 5–7 days and subsequently matured with 100 ng mL^−1^ lipopolysaccharide (LPS) in the presence of 100 ng mL^−1^ α-GalCer for 48–72h. Type 1 NKT cells were purified from PBMC from healthy donors by magnetic bead sorting using anti-type 1 NKT microbeads. Purified type 1 NKT cells were stimulated weekly with irradiated (50 Gy) α-GalCer loaded mature moDC in Yssel’s medium^58^ supplemented with 1% human AB serum, 0.05 mM β-ME, 100 IU mL^−1^ sodium penicillin, 100 μg mL^−1^ streptomycin sulfate, 2.0 mM L-glutamine, 10 IU mL^−1^ rhIL-7, 10 ng mL^−1^ rhIL-15.

Primary human Vγ9Vδ2-T cells were generated and expanded as described previously.^17^ In brief, Vγ9Vδ2-T cells were isolated from human PBMCs of healthy donors by magnetic bead sorting using anti-TRDV2 in combination with anti-mouse IgG microbeads. Purified Vγ9Vδ2-T cells were stimulated weekly with an irradiated (50 Gy) feeder mixture (PBMCs of two healthy human donors and JY cells (10:1 ratio)) in RPMI-1640 supplemented with 10% (v/v) FCS, 0.05 mM β-ME, 100 IU mL^−1^ sodium penicillin, 100 μg mL^−1^ streptomycin sulfate, 2.0 mM L-glutamine, 10 IU mL^−1^ rhIL-7, 10 ng mL^−1^ rhIL-15 and 50 ng mL^−1^ phytohaemagglutinin (PHA).

Expanded type 1 NKT and Vγ9Vδ2-T cells selected for experiments were >90% (>97% for mouse experiments) TRAV10^+^TRBV25-1^+^ and TRGV9^+^TRDV2^+^, respectively.

#### Type 1 NKT and Vγ9Vδ2-T cell stimulation assay

To evaluate induction of degranulation of type 1 NKT and Vγ9Vδ2-T cells, 1×10^5^ target cells (CCRF-CEM, MM1s.WT, MM.1s.CD1d, MOLM-13, NOMO-1, HeLa.WT, HeLa.CD1d, A-431) whether or not labeled with PBSE (CCRF-CEM only) were co-cultured with medium (negative control), VHH1D12 or bsTCE at the indicated concentration, and with 5×10^4^ type 1 NKT cells, 5×10^4^ Vγ9Vδ2-T cells or a mixture of 2.5×10^4^ type 1 NKT and 2.5×10^4^ Vγ9Vδ2-T cells for 4h in the presence of PE-labelled anti-CD107a, after which type 1 NKT ((PBSE^−^)CD3^+^CD45^+^TRGV9^−^TRAV-10^+^(CD1d(α-GalCer)-tetramer^+^)) and Vγ9Vδ2-T cells ((PBSE^−^)CD3^+^CD45^+^TRGV9^+^TRAV-10^-^((TRDV2^+^)(CD1d(α-GalCer)-tetramer^−/+^)) were analyzed for CD107a expression. Supernatants from 24h co-cultures of 1 × 10^5^ MM.1s.CD1d and 5×10^4^ type 1 NKT, Vγ9Vδ2-T cells or a 1:1 mixture thereof (ratio 1:1) with medium (negative control) or CD1d-Vδ2 bsTCE (50 nM) were analyzed for cytokine production using a cytometric bead array (CBA).

#### Diverse NKT cell stimulation and tetramer blocking assay

For the stimulation assays of the CD1d(sulfatide)-restricted TCR DP10.7 JRT3 transfectant, 1×10^5^ cells were co-cultured overnight ±1 × 10^5^ C1R.WT or C1R.CD1d cells and CD1d-Vδ2 bsTCE (1 μM), vehicle control (DMSO), sulfatide (25 μg mL^−1^) or a combination thereof. CD3^+^CD19^−^ cells were analyzed for CD69 expression.

CD1d(sulfatide) tetramer-PE was generated as described previously.^23^ For tetramer staining, endogenous or sulfatide-loaded CD1d tetramers were first incubated ± ∼10 times molar excess CD1d-Vδ2 bsTCE for 1h at room temperature. JRT3.DP10.7 cells and PBMC were then stained with tetramers and antibody cocktails for 1h at 4°C in the dark after which tetramer binding to CD3^+^(TRDV1^+^)7-AAD^-^ cells was assessed using flow cytometry.

For assessment of the capacity of CD1d-Vδ2 bsTCE to interfere with CD1d-sulfatide reactivity of diverse NKT cells in PBMC, healthy donor PBMC were enriched for CD1d(sulfatide)-reactive diverse NKT cells by magnetic bead sorting using CD1d(sulfatide) tetramer-PE in combination with anti-PE IgG microbeads (essentially as described^59^). The enriched cell fraction was then additionally stained with CD1d(sulfatide) tetramers pre-incubated ± 2 μM CD1d-Vδ2 bsTCE after which the percentage of CD1d(sulfatide)^+^ T cells was determined (within CD3^+^TRGV9^−^TRDV2^-^7-AAD^-^ cells; when present TRGV9^+^TRDV2^+^ cells were excluded from the analysis due to direct binding of the CD1d-Vδ2 bsTCE to these cells).

#### Cytotoxicity and effector cell proliferation assay

The ability of human type 1 NKT and Vγ9Vδ2-T cells to lyse target cells was determined by flow cytometry. 1×10^5^ target cells (CCRF-CEM (whether or not stained with PBSE), MOLM-13, NOMO-1 cells) were incubated with 5×10^4^ type 1 NKT, Vγ9Vδ2-T cells or a 1:1 mixture thereof with medium (negative control) or bsTCE at indicated concentration. After 16h incubation, cells were stained with an antibody cocktail, washed in annexin-V buffer and briefly incubated with annexin-V FITC, 7-AAD and flow cytometric counting beads, for subsequent flow cytometry analysis. Absolute number of living target cells (PBSE^+^ (CCRF-CEM cells only) CD3^-/dim^(CD7^+^)(CD33^+^)TRAV10^−^TRGV9^−^annexin V^−^ 7-AAD^-^) was determined according to manufacturer’s instructions. Specific lysis of target cells was calculated as follows; 100 - ((absolute cell number [condition]/absolute cell number [target cells only]) x 100).

To examine tumor growth control, and type 1 NKT and Vγ9Vδ2-T cell expansion, MM.1s.CD1d or MOLM-13 cells were incubated with medium (negative control) or CD1d-Vδ2 bsTCE (50 nM) and cultured for up to 7 days in the presence of human healthy donor derived type 1 NKT, Vγ9Vδ2-T cells or a 2:3 mixture thereof (E:T 1:10). Living target cells (CD3^-^(CD45^−/+^)TRAV-10^−^TRGV9^-^7-AAD^-^), type 1 NKT (CD3^+^(CD45^+^)TRAV-10^+^TRGV9^-^7-AAD^-^) and Vγ9Vδ2-T cells (CD3^+^(CD45^+^)TRAV-10^−^TRGV9^+^7-AAD^-^) were quantified by flow cytometric counting beads. Tumor growth and effector cell expansion factor was calculated by dividing the cell count after co-culture by the starting cell count at day 0.

#### PBMC cytotoxicity and proliferation assay

To examine B cell and monocyte lysis, tumor growth control, and type 1 NKT and Vγ9Vδ2-T cell degranulation and expansion in PBMC, 1×10^5^ healthy donor derived PBMC were incubated with or without 1×10^4^ target cells (MM.1s.CD1d or MOLM-13) and with medium (negative control) or CD1d-Vδ2 bsTCE (50 nM or concentration range) (and PE-labelled anti-CD107a for 16h culture only) and cultured up to 7 days.

Living target cells (B cells (CD3^−^CD19^+^CD33^−^CD45^+^7-AAD^-^), monocytes (CD3^−^CD14^+^CD19^−^CD33^+^CD45^+^7-AAD^-^), and MM.1s.CD1d (CD3^−^CD38^+^CD45^-^7-AAD^-^), and CD107a expression on type 1 NKT (CD3^+^CD45^+^TRGV9^−^TRDV2^−^TRAV10^+^CD1d(α-GalCer)-tetramer^+^7-AAD^-^) and Vγ9Vδ2-T cells (CD3^+^CD45^+^TRGV9^+^TRDV2^+^TRAV10^−^CD1d(α-GalCer)-tetramer^−/+^7-AAD^-^) were quantified/determined by flow cytometry counting beads after 16h. Specific lysis was calculated as described above. To determine tumor growth and effector cell expansion, living tumor cells (MM.1s.CD1d; CD3^−^CD38^+^CD45^-^7-AAD^-^, MOLM-13; CD3^−^CD33^++^CD45^dim^7-AAD^-^) and living type 1 NKT (CD3^+^(CD33^−^)CD45^+^TRGV9^−^TRDV2^−^TRAV10^+^CD1d(α-GalCer)-tetramer^+^7-AAD^-^) and Vγ9Vδ2-T cells (CD3^+^(CD33^−^)CD45^+^TRGV9^+^(TRDV2^+^)TRAV10^−^CD1d(α-GalCer)-tetramer^−/+^7-AAD^-^) were quantified by flow cytometric counting beads. Expansion factor was calculated by dividing the cell count after co-culture by the starting cell count.

To determine the ability of freshly isolated (untouched) Vγ9Vδ2-T cells to lyse either THP-1 tumor cells or freshly isolated (untouched) monocytes, Vγ9Vδ2-T cells and monocytes were isolated from healthy donor PBMC by magnetic bead sorting using a human γδ-T cell isolation kit and a pan monocyte isolation kit, respectively. 5×10^4^ target cells (monocytes (>65% CD14^+^ after isolation), THP-1 cells (stained with cell trace violet (CTV)) were incubated with 5×10^4^ Vγ9Vδ2-T cells (>75% of CD3^+^ cells after isolation) with medium (negative control) or 1 nM CD1d-Vδ2 bsTCE. After 24h incubation, cells were stained with an antibody cocktail, washed and briefly incubated with 7-AAD, for subsequent flow cytometry analysis. The percentage of 7-AAD^+^ monocytes (CD3^−^CD14^+^TRGV9^-^) and THP-1 cells (CD3^−^TRGV9^−^CTV^+^) was determined.

#### Humanization and binding of pre-existing anti-VHH antibodies

*In silico* humanization was performed by Lonza (Lonza Biologics) via selection of human antibody germline sequences similar to monovalent VHH (CD1d VHH1D12; IMGT: IGHV3-66∗01, Vδ2 VHH5C8; IMGT: IGHV3-23∗01), identification of VHH critical residues, and complementarity-determining region (CDR) grafting and substitution of mismatched residues between parental and acceptor framework regions, similar as described previously.^60^ Sequences were analyzed for potential issues based on Ag binding, protein stability, function and sequence liabilities using a structural model. Epibase screening (Lonza) was used to screen humanized sequences for predicted T helper epitopes that may contribute to immunogenicity and to calculate an immunogenicity risk (HLA-DRB1-binding) score. For each monovalent VHH, 10 variants were generated and functionally evaluated as described above. The relative percentage of binding of the variants compared to the WT protein was calculated by dividing MF [condition] by MF [WT VHH] multiplied by 100. To generate humanized CD1d-Vδ2 bsTCE (CD1d-Vδ2 hu-bsTCE), CD1d VHH (clone VHH1D12.Q1E) was genetically linked by a G_4_S-linker to Vδ2 TCR (clone VHH5C8.variant 1, selected based on a combination of predicted protein manufacturing characteristics, human-ness and immunogenicity risk) and expressed in *P. pastoris* (Validogen Gmbh, Graz, Austria). VHH1D12.Q1E (glutamic acid substitution of glutamine at position 1; a residue that was shown not to interact with CD1d nor type 1 NKT TCR)^23^ was used instead of VHH1D12 WT due to reduced and inconsistent leader peptide cleavage when the Q1 containing bsTCE was produced in the *P. pastoris* strain (data not shown). HLA-DRB1-binding score of the monovalent binding arms of the CD1d-Vδ2 bsTCE (i.e. CD1d VHH1D12 and Vδ2-TCR VHH5C8) was compared to the HLA-DRB1-binding score of therapeutic human IGHV.^61^ CD1d-Vδ2 hu-bsTCE was functionally evaluated as described above.

The frequency of pre-existing human anti-VHH antibodies in serum was determined via an Ag binding test by Sanquin Blood Supply foundation. CD1d-Vδ2 bsTCE and CD1d-Vδ2 hu-bsTCE were labeled with radio-active iodine (I^125^) and purified. I^125^-labeled bsTCE (∼0.5 ng per test) was subsequently incubated with 1 μL healthy donor human serum (1:50 diluted in 0.3% PBS-BSA) and possible IgG-VHH complexes were then captured onto protein-G coated Sepharose beads. After overnight incubation and extensive washing, radioactivity of sepharose-beads was measured by a gamma counter. Specificity of the test was confirmed by inhibition of the signal by adding an excess of unlabeled bsTCE (100 ng/test, ∼200 times excess). Percentage of binding was calculated by dividing the number of disintegrations measured on the sepharose-bound radiolabeled bsTCE in the experimental condition by the number of disintegrations found in the total input of radiolabeled bsTCE. The cut-off point for positivity was calculated on the mean percentage binding (after removal of outliers using iterative Grubbs) plus 1.645 SD.

#### Patient-derived MM, AML, and CLL samples

Using multicolor flow cytometry, CD1d geometric median fluorescence (MF) was determined on MM (CD3^−^CD19^−/+^CD38^+^CD45^-/dim^CD56^−/+^(CD81^−/+^)(CD117^−/+^)CD138^+^), AML (CD3^−^CD33^−/+^CD34^−/+^CD45^dim/+^CD56^−/+^CD117^−/+^HLA-DR^dim/+^) and CLL cells (CD3^−^CD5^+^CD19^+^). Geometric MF index (MFI) was calculated by dividing MF [stained tumor cells] by MF [unstained tumor cells or CD1d^−^ population]. T cell percentage (CD3^+^CD45^+^) of mononuclear cells, type 1 NKT percentage (CD3^+^CD45^+^TRAV10^+^CD1d(α-GalCer)-tetramer^+^/TRBV25-1^+^) of CD3^+^ cells and Vγ9Vδ2-T percentage (CD3^+^CD45^+^TRGV9^+^TRDV2^+^) of CD3^+^ cells was determined using multicolor flow cytometry. For autologous and allogeneic type 1 NKT and Vγ9Vδ2-T cell degranulation, and cytotoxicity toward patient MM, monocytic AML and CLL cells, 1×10^5^ thawed BMMC (MM and AML) or PBMC (CLL ± OCH (4h, 100 ng mL^−1^) or thapsigargin (6h, 30 μM) pre-incubation) were cultured with medium (negative control) or CD1d-Vδ2 hu-bsTCE (50 nM) in the presence or absence of 5×10^4^ type 1 NKT, Vγ9Vδ2-T cells or a 1:1 mixture thereof for 16h in the presence of PE-labelled anti-CD107a, after which type 1 NKT (7-AAD^−^CD3^+^ (CD19^−^)(CD33^−^)CD45^+^(CD138^-^)TRGV9^−^TRAV-10^+^CD1d(α-GalCer)-tetramer^+^) and Vγ9Vδ2-T cells (7-AAD^−^CD3^+^(CD19^−^)(CD33^−^)CD45^+^(CD138^-^)TRGV9^+^TRAV-10^-^((TRDV2^+^)(CD1d(α-GalCer)-tetramer^−/+^)) were analyzed for CD107a expression. Live MM (7-AAD^−^CD3^−^(CD19^−^)CD45^dim/−^CD38^+^(CD56^−/+^)CD138^+^), monocytic AML (7-AAD^−^CD3^−^CD33^dim/+^(CD34^+^)CD45^dim/+^(CD56^+^CD117^+^)HLA-DR^dim/+^) and CLL cells (7-AAD^−^CD3^−^CD5^+^CD19^+^CD45^+^) were quantified by flow cytometry counting beads. Specific lysis was calculated as described above. Supernatants were collected and analyzed for cytokine production by CBA.

#### Functional analyses of NHP surrogate bsTCE

To examine cross-reactivity of CD1d-Vδ2 bsTCE toward NHP CD1d and Vγ9Vδ2-TCR, monovalent VHHs (CD1d VHH1D12 and VHH1D22^23^ and a previously generated panel of Vγ9Vδ2-TCR VHHs^42^) were biotinylated using NHS-D-biotin according to the manufacturer’s protocol; 0.2–0.5 x 10^6^ human PBMC (positive control), *M. fascicularis* (origin China, Mauritius, and Vietnam) PBMC or *M. mulatta* PBMC (CITox, Miserey, France) were subsequently incubated with indicated VHH (2 μg mL^−1^, 120–150 nM) and antibody cocktail (CD20-FITC, TRGV9-PE, CD11b-PEcy7, streptavidin-APC and TCR pan-γδ-BV421). CD11b^+^ CD20^−^ TRGV9^-^ pan-γδ^-^ cells and CD11b^−^ CD20^−^ TRGV9^+^ pan-γδ^+/dim^ were analyzed by flow cytometry for CD1d VHH and Vγ9Vδ2-TCR VHH binding respectively as determined by streptavidin-APC fluorescence.

To be able to assess safety, PK and PD parameters in NHP, a surrogate bispecific molecule was generated using sequence information from an NHP cross-reactive mouse antibody (clone 7A5, kind gift from dr. D. Kabelitz) directed to the Vγ9 chain of the TCR, coupled to the cross-reactive CD1d specific VHH1D22 (CD1d VHH1D22-scFv7A5, referred to as CD1d-Vγ9 bsTCE) and produced by UPE. Bispecific molecule CD1d VHH1D12-scFv7A5 (control Vγ9 bsTCE), which lacks cross-reactivity toward NHP CD1d, was generated as a control and produced by UPE. Binding of the various bispecific molecules to CD1d and Vγ9Vδ2-TCR was analyzed at indicated concentrations by flow cytometry as described above. For binding to monocytes and Vγ9-T cells in PBMC (human or NHP), 0.25–1×10^5^ cells were incubated with indicated concentrations of biotinylated bsTCE for ∼45 min at 4°C, followed by extensive washing, incubation with Fc-block and stained with CD11b-PEcy7, streptavidin-APC and CD3-AF700. CD1d and Vγ9-TCR binding was determined by streptavidin-APC MF on monocytes (CD11b^++^(CD20^−^)) and Vγ9-T cells (CD3^+^CD11b^-^streptavidin-APC^+^). Relative percentage of binding was calculated as described above. To evaluate induction of degranulation of Vγ9-T cells, thawed human and NHP primate PBMC (0.25–1×10^5^ per condition) were incubated with rhIL-2 (50 IU/mL) for 24h to recover followed by incubation with or without CD1d-Vδ2 bsTCE, CD1d-Vγ9 bsTCE or control Vγ9 bsTCE (concentration range) for an additional 24h in the presence of PE-labelled anti-CD107a, after which Vγ9-T cells (CD3^+^ CD11b^−^ TRGV9^+^) were analyzed for CD107a expression.

### Quantification and statistical analysis

Sample size for the MM *in vivo* experiments was determined by using a two-sided, two-sample equal-variance t-test (assuming a population mean difference of 0.25 with an s.d. of 0.15 and with a significance level (α) of 0.050 with >80% power) and previous experience. Sample size for the AML and T-ALL *in vitro* experiments was estimated based on previous experience with these tumor cell lines. Mice were randomly assigned to treatment groups. No statistical method was used to predetermine sample size for the *in vitro* experiments. For *in vitro* experiments each n represents an independent experiment with, when applicable, primary type 1 NKT and Vγ9Vδ2-T cell lines, and PBMC obtained from individual healthy donors. For experiments with patient MM and AML BMMC and CLL PBMC each n represents an individual patient. For *in vivo* experiments each n represents an individual animal. No data were excluded from the analyses. The experiments were not randomized and the investigators were not blinded to allocation during the experiments and outcome assessment.

For data with one variable and two groups, a two-tailed paired/unpaired t-test was used to calculate the p value. For data with one variable and multiple groups, a one-way analysis of variance (ANOVA) with Tukey’s multiple comparisons test to calculate the multiplicity-adjusted p value was used. For data with two variables, a two-way ANOVA with either Tukey’s or Šídák’s multiple comparisons test to calculate the multiplicity-adjusted p value was used. Dose–response curves, half maximal effective concentration (EC_50_) was calculated using non-linear regression (agonist versus normalized response for the binding experiments and agonist versus response for the remaining experiments). To determine the relationship between CD1d-expression and cell lysis, linear regression was used to fit the line through the data points, determine goodness of fit (R2) and calculate the p value. Survival data was analyzed with Kaplan–Meier survival curves and the log rank test (two-tailed p values) was used to calculate statistically significant differences. Statistical details can be found in the figure legend. Statistical analysis was performed using Prism v.9.1.0 (GraphPad Software).

## Data Availability

All data reported in this paper will be shared by the lead contact upon request. This paper does not report original code. Any additional information required to reanalyze the data reported in this paper is available from the lead contact upon request.

## Supplemental information

Document S1. Figures S1–S7 and Tables S1–S4

Document S2. Article plus supplemental information
