# Supplementary material for: A bispecific T cell engager recruits both type 1 NKT and Vγ9Vδ2-T cells for the treatment of CD1d-expressing hematological malignancies
Source: Cell Rep Med. 2023 Mar 2;4(3):100961. doi: 10.1016/j.xcrm.2023.100961 (PMC10040383; doi:10.1016/j.xcrm.2023.100961)
Supplement: Document S1. Figures S1–S7 and Tables S1–S4 [file mmc1.pdf]

Cell Reports Medicine, Volume *4*

Supplemental information

A bispecific T cell engager recruits both type 1 NKT and Vγ9Vδ2-T cells for the treatment of CD1d-expressing hematological malignancies

Roeland Lameris, Jurjen M. Ruben, Victoria Iglesias-Guimarais, Milon de Jong, Myrthe Veth, Fleur S. van de Bovenkamp, Iris de Weerdt, Arnon P. Kater, Sonja Zweegman, Sjeng Horbach, Thilo Riedl, Benjamin Winograd, Rob C. Roovers, Anton E.P. Adang, Tanja D. de Gruijl, Paul W.H.I. Parren, and Hans J. van der Vliet

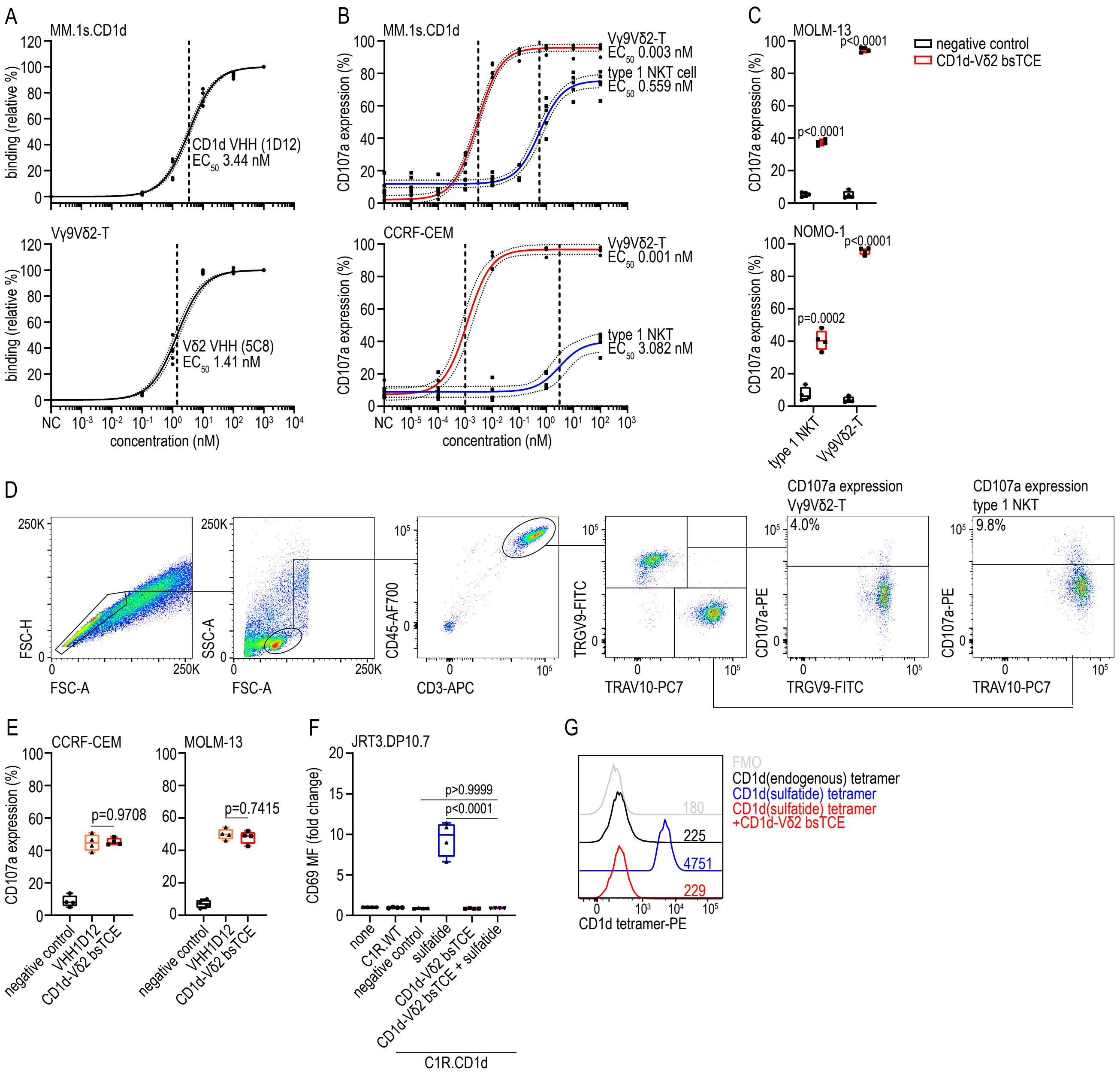

Figure S1. CD1d-Vδ2 bsTCE activates both type 1 NKT and Vγ9Vδ2-T cells (related to figure 1).

(A) Relative binding of a concentration range of CD1d VHH (1D12) and Vδ2 VHH (5C8) to MM.1s.CD1d (n=5) and Vγ9Vδ2-T cells (n=5), detected by rabbit-anti-llama (RαL)-iFluor488.

(B,C) CD107a expression on type 1 NKT and Vγ9Vδ2-T cells after 4h co-culture of 1:1 mixed effector cells and either MM.1s.CD1d (B, n=5), CCRF-CEM (B, n=4), MOLM-13 (C, n=4) or NOMO-1 (C, n=4) ± concentration range (B) or 100 nM (C) CD1d-Vδ2 bsTCE.

(D) Exemplifying gating strategy used for type 1 NKT and Vγ9Vδ2-T cell CD107a expression analyses (here after co-culture with MM.1s.CD1d).

(E) CD107a expression on type 1 NKT after 4h co-culture with either CCRF-CEM (n=4) or MOLM-13 (n=4) ± 100 nM VHH1D12 or CD1d-Vδ2 bsTCE.

(F) Fold change in CD69 expression on the CD1d(sulfatide) restricted diverse NKT cell line JRT3.DP10.7 after overnight co-culture with either C1R.WT or C1R.CD1d ± 25 μg ml^{-1} sulfatide, 1 μM CD1d-Vδ2 bsTCE or a combination thereof (n=4).

(G) Histograms depicting the binding of endogenous- or sulfatide-loaded CD1d tetramers pre-incubated ± CD1d-Vδ2 bsTCE (~10 times molar excess) to JRT3.DP10.7 cells. The number in each histogram indicates the median fluorescence.

Negative control (NC) indicates PBS (A) or medium control (B,C,E,F). Box and whisker plots indicate the median, 25th-75th percentiles and minimum-maximum. Non-linear regression with 95% confidence bands (dotted lines) and EC_{50s} (dashed lines) (A,B). Two-way ANOVA with Šídák multiple comparisons test (C), one-way ANOVA with Tukey multiple comparisons test (E,F).

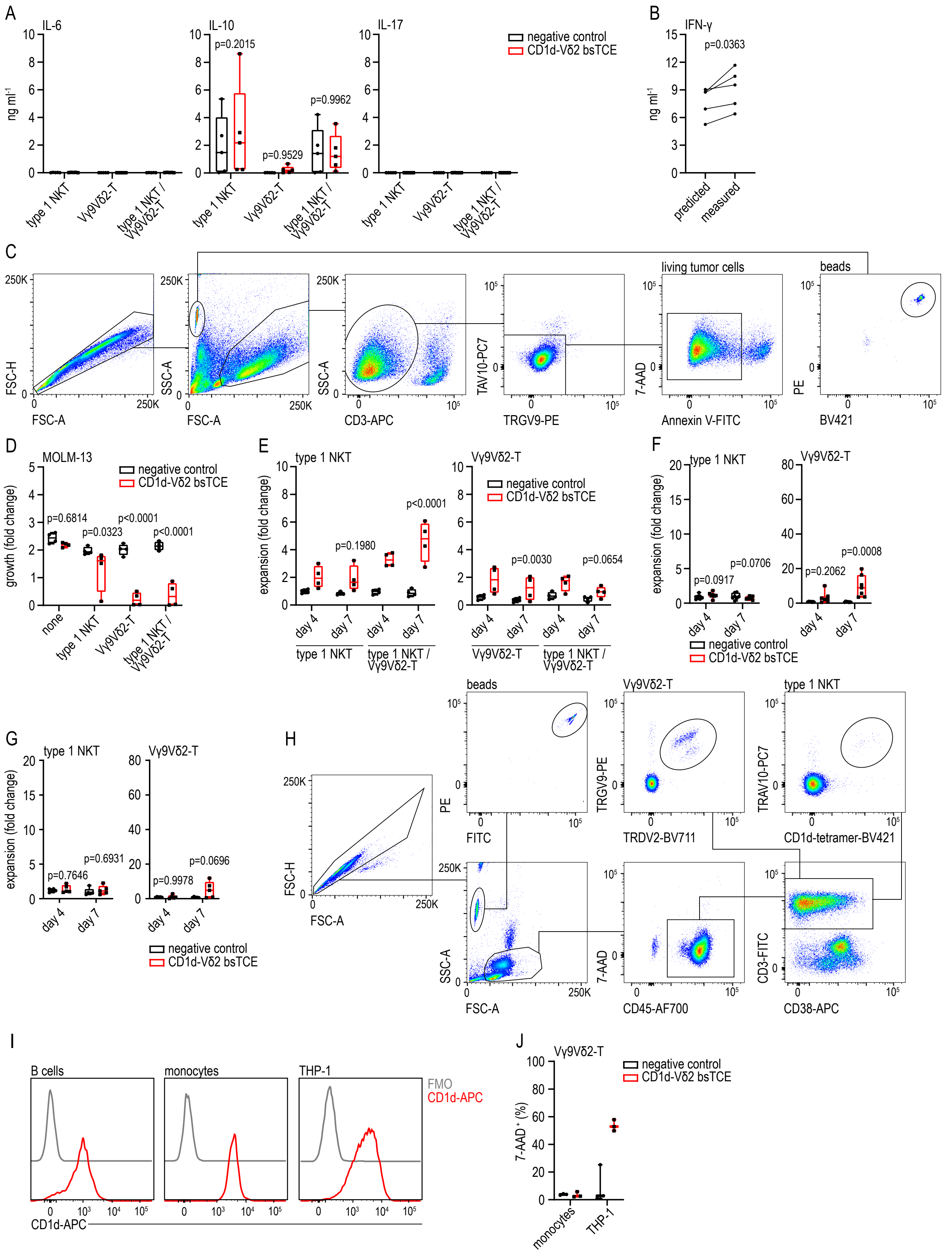

A
IL-6
IL-10
IL-17
ng ml-1
p=0.2015
p=0.9529
p=0.9962
type 1 NKT
Vγ9Vδ2-T
type 1 NKT / Vγ9Vδ2-T
negative control
CD1d-Vδ2 bsTCE
B
IFN-γ
p=0.0363
predicted
measured
C
living tumor cells
beads
FSC-H
FSC-A
SSC-A
CD3-APC
TAV10-PC7
TRGV9-PE
7-AAD
Annexin V-FITC
PE
BV421
D
MOLM-13
growth (fold change)
p=0.6814
p=0.0323
p<0.0001
p<0.0001
none
E
expansion (fold change)
p=0.1980
p<0.0001
p=0.0030
p=0.0654
day 4
day 7
F
p=0.0917
p=0.0706
p=0.2062
p=0.0008
G
p=0.7646
p=0.6931
p=0.9978
p=0.0696
H
FITC
TRGV9-PE
TRDV2-BV711
TRAV10-PC7
CD1d-tetramer-BV421
CD45-AF700
CD3-FITC
CD38-APC
I
B cells
monocytes
THP-1
FMO
CD1d-APC
J
7-AAD+ (%)
monocytes

Figure S2. CD1d-Vδ2 bsTCE triggers both type 1 NKT and Vγ9Vδ2-T cell cytokine production, expansion and lysis of CD1d-expressing target cells (related to figure 2 and 3).

(A,B) Cytokine secretion by type 1 NKT, Vγ9Vδ2-T cells or a 1:1 mixture thereof after 24h co-culture with MM.1s.CD1d cells ± 50 nM CD1d-Vδ2 bsTCE (n=5). Predicted indicates the amount of IFN-γ as expected based on the concentrations of IFN-γ in co-cultures containing either effector cell population alone, measured indicates the actual IFN-γ amount detected in the co-culture (B).

(C) Exemplifying gating strategy used to analyze type 1 NKT and/or Vγ9Vδ2-T cell cytotoxicity towards tumor cells (here MOLM-13).

(D,E) Fold growth of MOLM-13 cells (D, n=4, day 7) and fold expansion of type 1 NKT and Vγ9Vδ2-T cells (type 1 NKT / Vγ9Vδ2-T indicates mixed effector cells, ratio 2:3) after up to 7 day (co-)culture ± 50 nM CD1d-Vδ2 bsTCE (E:T ratio of 1:10) (E, n=4).

(F,G) Fold expansion of type 1 NKT and Vγ9Vδ2-T cells after up to 7 day (co-)culture of PBMC (F, n=7) or PBMC and MOLM-13 (G, n=5) (ratio 10:1) ± 50 nM CD1d-Vδ2 bsTCE.

(H) Exemplifying gating strategy used to analyze type 1 NKT and/or Vγ9Vδ2-T cell frequencies (here in PBMC).

(I) Exemplary histograms depicting CD1d expression on B cells and monocytes in PBMC, and THP-1 cells. FMO, fluorescence minus one.

(J) Lysis of purified (untouched) monocytes and THP-1 cells after 24h co-culture with purified (untouched) Vγ9Vδ2-T cells (E:T ratio 1:1) ± 1 nM CD1d-Vδ2 bsTCE (n=3).

Negative control indicates medium control. Box and whisker plots indicate the median, 25th-75th percentiles and minimum-maximum. Two-way ANOVA with Šídák (A,D,E,F,G) multiple comparisons test, two tailed paired T-test (B).

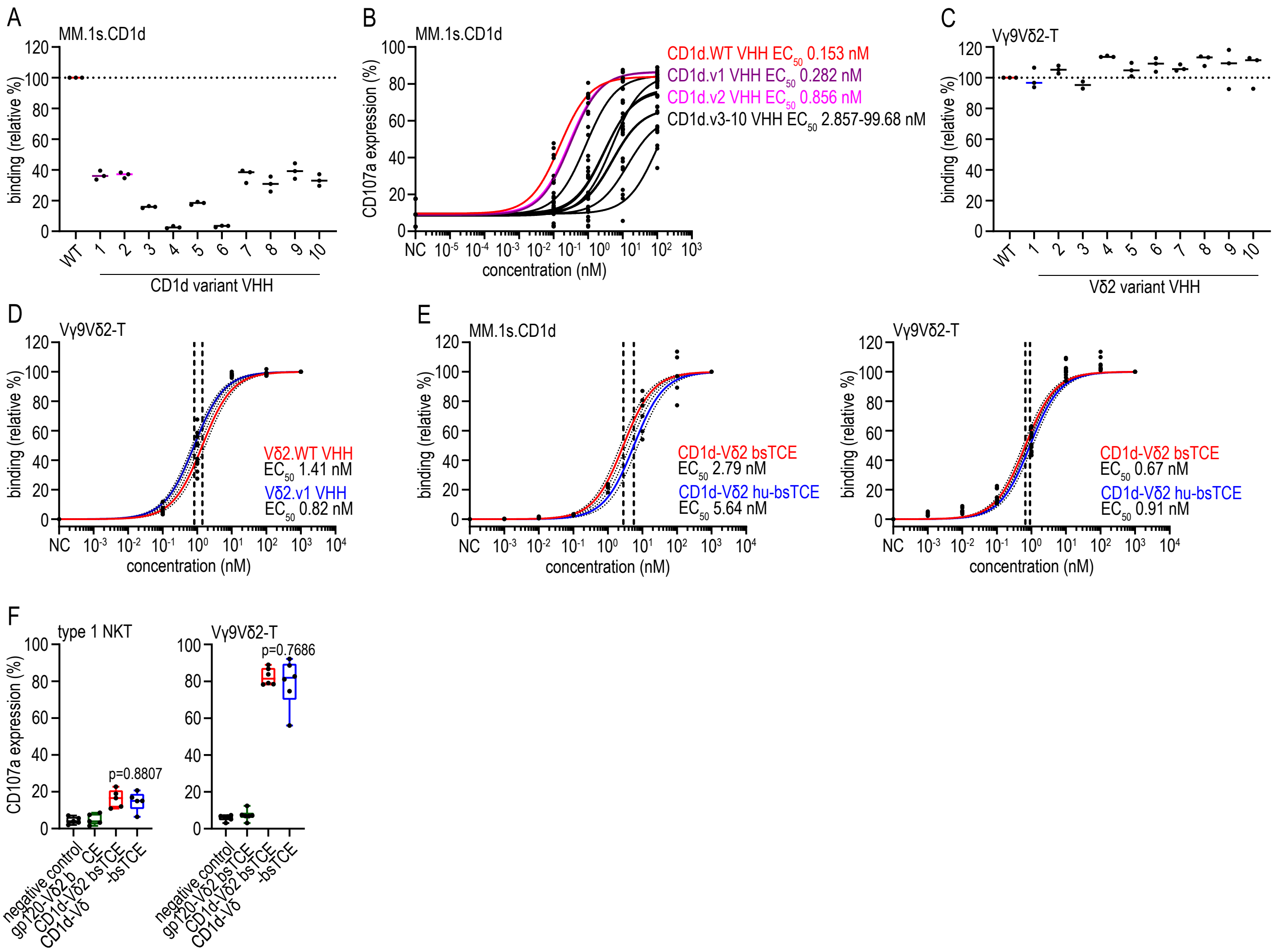

Figure S3. Humanization of CD1d-Vδ2 bsTCE does not affect functionality (related to figure 4).

(A) Relative binding of 100 nM VHH1D12 (WT) or humanized variant VHHs to MM.1s.CD1d (n=3), detected by GαL-FITC.

(B) CD107a expression on type 1 NKT cells after 4h co-culture with MM.1s.CD1d ± concentration range of indicated VHHs (n=3).

(C-E) Relative binding of 100 nM (C) or concentration range (D,E) of indicated VHHs or bsTCEs to Vγ9Vδ2-T cells (C, n=3; D, n=5; E, n=6) or MM.1s.CD1d (E, n=3), detected by GαL-FITC or RαL-iFluor488.

(F) CD107a expression on type 1 NKT and Vγ9Vδ2-T cells after 16h co-culture of PBMC and MOLM-13 cells (ratio 10:1) ± 50 nM bsTCE (type 1 NKT, n=5; Vγ9Vδ2-T, n=6).

Negative control (NC) indicates PBS (D,E) or medium control (B,F). Box and whisker plots indicate the median, 25th-75th percentiles and minimum-maximum. Non-linear regression with 95% confidence bands (dotted lines) and EC_{50s} (dashed lines) (B,D,E). One-way ANOVA with Tukey multiple comparisons test (F).

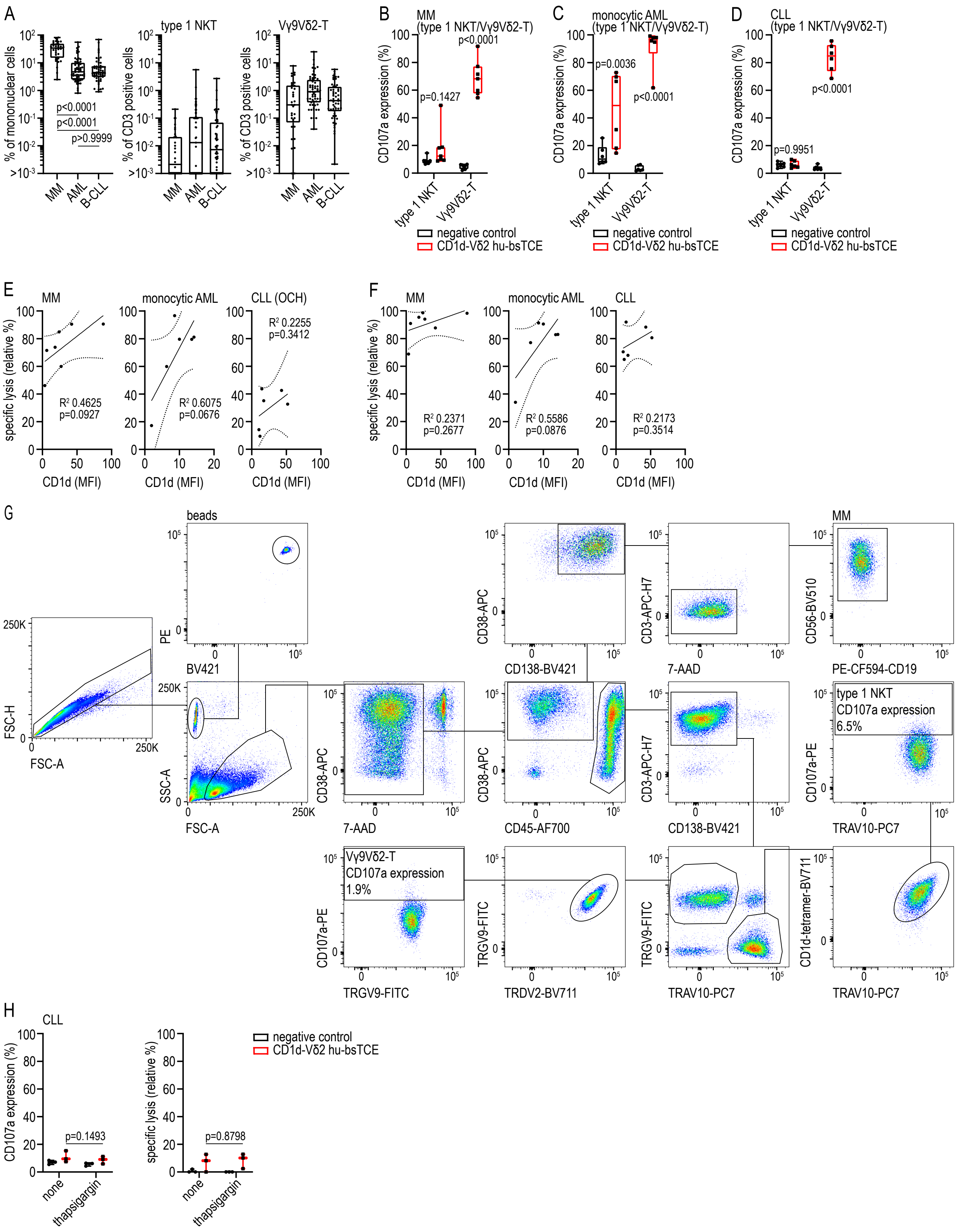
A
% of mononuclear cells
p<0.0001
p<0.0001
p>0.9999
MM
AML
B-CLL
type 1 NKT
% of CD3 positive cells
Vγ9Vδ2-T
B
MM
(type 1 NKT/Vγ9Vδ2-T)
CD107a expression (%)
p=0.1427
p<0.0001
C
monocytic AML
(type 1 NKT/Vγ9Vδ2-T)
p=0.0036
p<0.0001
D
CLL
(type 1 NKT/Vγ9Vδ2-T)
p=0.9951
p<0.0001
type 1 NKT
Vγ9Vδ2-T
negative control
CD1d-Vδ2 hu-bsTCE
E
MM
monocytic AML
CLL (OCH)
specific lysis (relative %)
R² 0.4625
p=0.0927
R² 0.6075
p=0.0676
R² 0.2255
p=0.3412
CD1d (MFI)
F
MM
monocytic AML
CLL
R² 0.2371
p=0.2677
R² 0.5586
p=0.0876
R² 0.2173
p=0.3514
G
beads
PE
BV421
FSC-H
FSC-A
SSC-A
CD38-APC
7-AAD
CD138-BV421
CD45-AF700
CD3-APC-H7
MM
CD56-BV510
PE-CF594-CD19
type 1 NKT
CD107a expression
6.5%
CD107a-PE
TRAV10-PC7
Vγ9Vδ2-T
CD107a expression
1.9%
TRGV9-FITC
TRDV2-BV711
CD1d-tetramer-BV711
H
CLL
CD107a expression (%)
p=0.1493
specific lysis (relative %)
p=0.8798
none
thapsigargin
negative control
CD1d-Vδ2 hu-bsTCE

Figure S4. Type 1 NKT and Vγ9Vδ2-T cell frequency in patient-derived MM, monocytic AML and CLL mononuclear cells, and correlation of CD1d-Vδ2 hu-bsTCE-induced effector cell cytotoxicity with CD1d expression levels (related to figure 5).

(A) Total T lymphocyte (MM 34.86±21.04%; AML 8.60±10.32%; CLL 8.60±12.24% (mean±SD of living mononuclear cells)), type 1 NKT cell (MM 0.02±0.04%; AML 0.20±0.74%; CLL: 0.11±0.39% (mean±SD of total T cells)) and Vγ9Vδ2-T cell (1.10±1.71%; AML 2.14±3.84%; CLL 1.01±1.31% (mean±SD of total T cells)) frequency in patient MM BMMC (n=34), AML BMMC (n=59) and CLL PBMC (n=54).

(B-D) CD107a expression on allogeneic type 1 NKT and Vγ9Vδ2-T cells after a 16h co-culture of patient MM BMMC (B, n=7), AML BMMC (C, n=6) or CLL PBMC (D, n=6) and a 1:1 mix of type 1 NKT and Vγ9Vδ2-T cells (effector:BMMC/PBMC ratio 1:2) ± 50 nM CD1d-Vδ2 hu-bsTCE.

(E,F) Correlation of CD1d expression and type 1 NKT cell-induced (E) or Vγ9Vδ2-T cell-induced (F) specific lysis of patient MM, monocytic AML and CLL (± pre-incubation with 100 ng ml^{-1} OCH for 4h) after 16h co-culture of BMMC (MM, n=7; monocytic AML, n=6) or PBMC (CLL, n=6) (effector:BMMC/PBMC ratio of 1:2) plus 50 nM CD1d-Vδ2 hu-bsTCE.

(G) Exemplifying gating strategy used for type 1 NKT and Vγ9Vδ2-T cell CD107a expression analyses and quantification of living tumor cells analyses (here co-culture of patient MM BMMC and mixed type 1 NKT and Vγ9Vδ2-T cells).

(H) CD107a expression on allogeneic type 1 NKT cells and specific lysis of patient CLL cells (n=3) after a 16h co-culture of PBMC, ± 6h pre-incubation with 30 nM thapsigargin, and type 1 NKT cells (effector:PBMC ratio 1:2) ± 50 nM CD1d-Vδ2 hu-bsTCE.

Negative control indicates medium control. Box and whisker plots indicate the median, 25th-75th percentiles and minimum-maximum. One-way ANOVA with Tukey multiple comparisons test (A) or two-way ANOVA with Šídák multiple comparisons test (B-D,H). Linear regression with 95% confidence bands (dotted lines) (E,F).

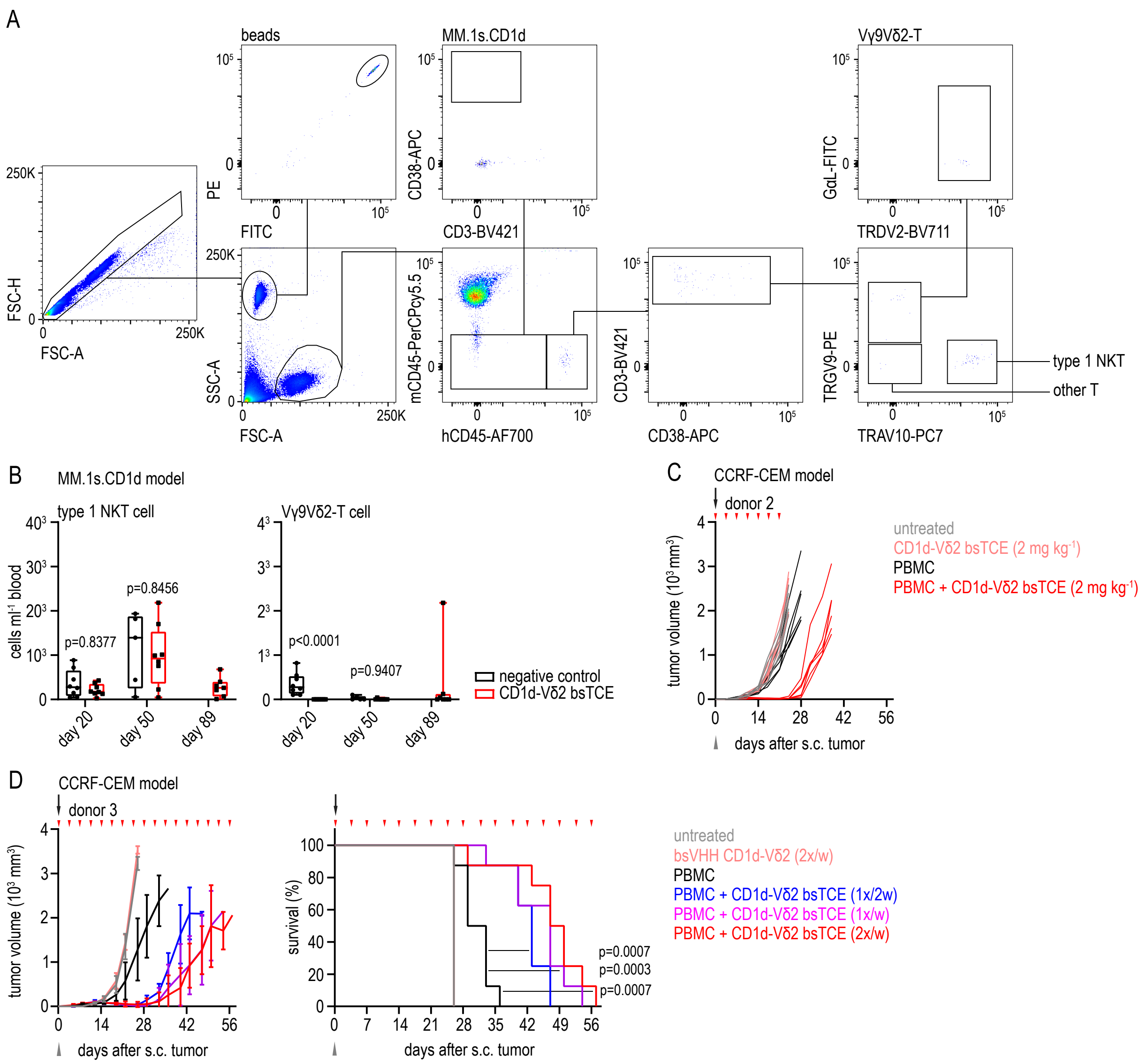

Figure S5. CD1d-Vδ2 bsTCE induces type 1 NKT and Vγ9Vδ2-T cell mediated tumor protection *in vivo* which improves survival (related to figure 6).

(A) Exemplifying gating strategy used to analyze *in vivo* type 1 NKT and Vγ9Vδ2-T cell frequencies in peripheral blood.

(B) Peripheral blood human type 1 NKT and Vγ9Vδ2-T cell frequency in mice engrafted with MM.1s.CD1d and infused with a 1:1 mix of type 1 NKT/Vγ9Vδ2-T cells (10^7 total cells, days 7, 14, 21) plus twice weekly i.p PBS (negative control) or CD1d-Vδ2 bsTCE (100 μg) (n=8).

(C) Tumor volume (mm^3) in individual mice of s.c. engrafted CCRF-CEM (10^7 cells) mixed with PBS (untreated, n=4) or healthy donor PBMC ($5x10^6$ cells, n=6) plus twice weekly i.p. PBS or CD1d-Vδ2 hu-bsTCE (indicated dose, up to day 21).

(D) Mean ±SD tumor volume (mm^3) and survival of mice s.c. engrafted with CCRF-CEM (10^7 cells) mixed with PBS (untreated, n=4) or healthy donor PBMC (10^7 cells, n=8) plus i.p. PBS or CD1d-Vδ2 hu-bsTCE (0.2 mg kg^{-1} at indicated interval).

Grey arrowheads: tumor inoculation; black arrows: PBS or effector cell injection; red arrowheads: PBS or bsTCE infusion. The box and whisker plots indicate the median, 25th-75th percentiles and minimum-maximum. Two-way ANOVA with Šídák multiple comparisons test (B). Log-rank test, two-tailed p-values (D).

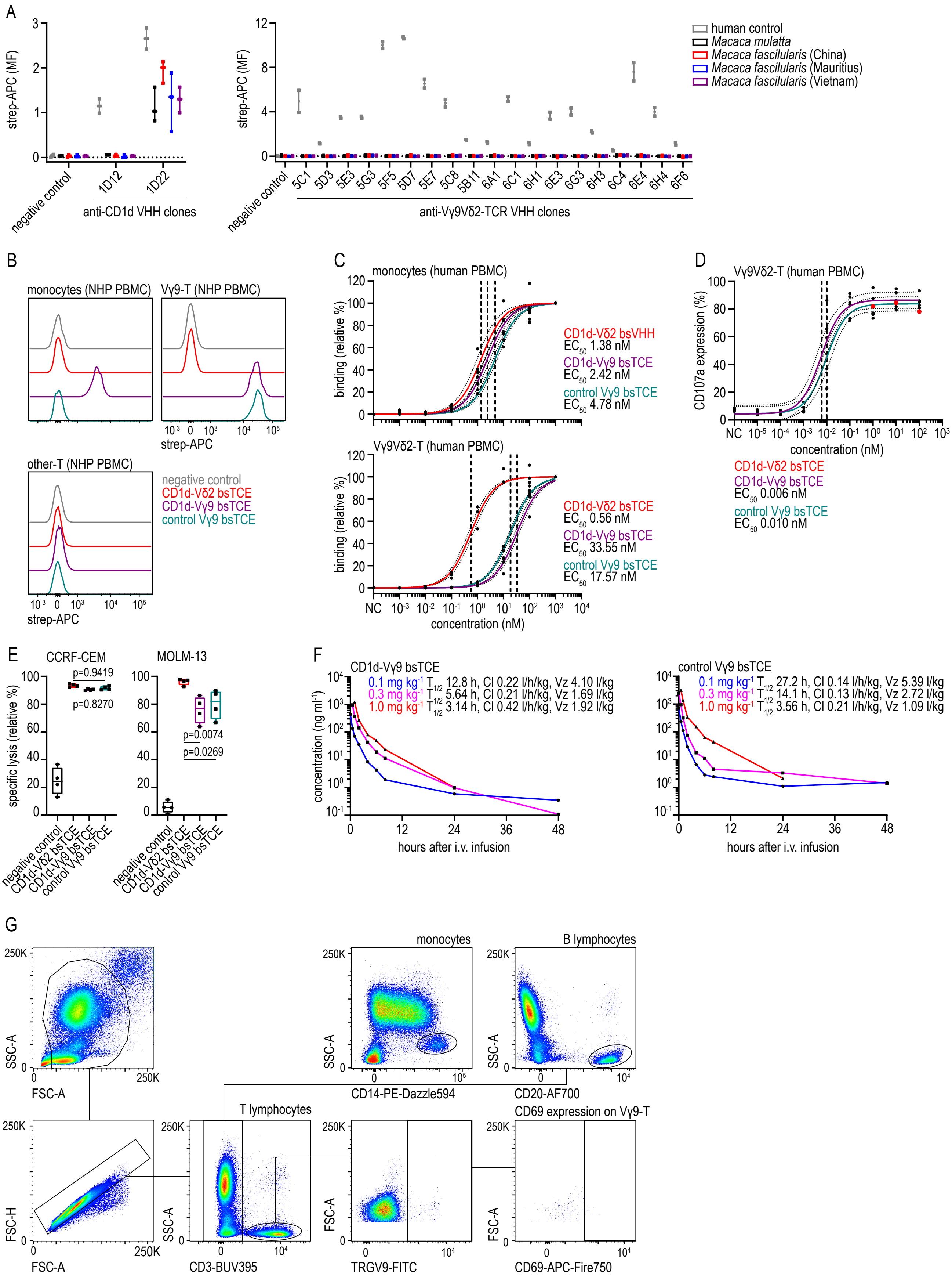
A
strep-APC (MF)
negative control
1D12
1D22
anti-CD1d VHH clones
5C1 5D3 5E3 5G3 5F5 5D7 5E7 5C8 5B11 6A1 6C1 6H1 6E3 6G3 6H3 6C4 6E4 6H4 6F6
anti-Vγ9Vδ2-TCR VHH clones
human control
Macaca mulatta
Macaca fascilularis (China)
Macaca fascilularis (Mauritius)
Macaca fascilularis (Vietnam)
B
monocytes (NHP PBMC)
Vγ9-T (NHP PBMC)
other-T (NHP PBMC)
strep-APC
negative control
CD1d-Vδ2 bsTCE
CD1d-Vγ9 bsTCE
control Vγ9 bsTCE
C
monocytes (human PBMC)
binding (relative %)
CD1d-Vδ2 bsVHH
EC50 1.38 nM
CD1d-Vγ9 bsTCE
EC50 2.42 nM
control Vγ9 bsTCE
EC50 4.78 nM
Vγ9Vδ2-T (human PBMC)
CD1d-Vδ2 bsTCE
EC50 0.56 nM
CD1d-Vγ9 bsTCE
EC50 33.55 nM
control Vγ9 bsTCE
EC50 17.57 nM
concentration (nM)
D
Vγ9Vδ2-T (human PBMC)
CD107a expression (%)
concentration (nM)
CD1d-Vδ2 bsTCE
CD1d-Vγ9 bsTCE
EC50 0.006 nM
control Vγ9 bsTCE
EC50 0.010 nM
E
CCRF-CEM
MOLM-13
specific lysis (relative %)
p=0.9419
p=0.8270
p=0.0074
p=0.0269
negative control
CD1d-Vδ2 bsTCE
CD1d-Vγ9 bsTCE
control Vγ9 bsTCE
F
CD1d-Vγ9 bsTCE
0.1 mg kg-1 T1/2 12.8 h, Cl 0.22 l/h/kg, Vz 4.10 l/kg
0.3 mg kg-1 T1/2 5.64 h, Cl 0.21 l/h/kg, Vz 1.69 l/kg
1.0 mg kg-1 T1/2 3.14 h, Cl 0.42 l/h/kg, Vz 1.92 l/kg
concentration (ng ml-1)
hours after i.v. infusion
control Vγ9 bsTCE
0.1 mg kg-1 T1/2 27.2 h, Cl 0.14 l/h/kg, Vz 5.39 l/kg
0.3 mg kg-1 T1/2 14.1 h, Cl 0.13 l/h/kg, Vz 2.72 l/kg
1.0 mg kg-1 T1/2 3.56 h, Cl 0.21 l/h/kg, Vz 1.09 l/kg
hours after i.v. infusion
G
SSC-A
FSC-A
FSC-H
monocytes
B lymphocytes
CD14-PE-Dazzle594
CD20-AF700
T lymphocytes
CD3-BUV395
TRGV9-FITC
CD69 expression on Vγ9-T
CD69-APC-Fire750

Figure S6. CD1d-Vγ9 bsTCE binds to and activates NHP Vγ9-T cells and has a short plasma half-life (related to figure 7).

(A) Binding of biotinylated CD1d VHH to monocytes and binding of biotinylated Vγ9/Vδ2-TCR VHHs to Vγ9-T cells (120-150 nM) (human n=2; NHP n=3) in PBMC, detected by streptavidin(strep)-APC. MF, median fluorescence.

(B) Representative histogram showing binding of indicated biotinylated bsTCE (1000 nM) to NHP monocytes, Vγ9-T and other-T cells (in PBMC) detected by streptavidin-APC.

(C) Relative binding of concentration range of indicated biotinylated bsTCE to human monocytes and Vγ9Vδ2-T cells (in PBMC) detected by streptavidin-APC (n=3).

(D) CD107a expression on human Vγ9Vδ2-T cells after 24h culture of human PBMC ± a concentration range of indicated bsTCE (n=3).

(E) Specific lysis of CCRF-CEM and MOLM-13 cells after 16h co-culture with human Vγ9Vδ2-T cells (E:T ratio of 1:2) ± 50 nM indicated bsTCE (n=4).

(F) Plasma half-life ($T_{1/2}$), Clearance (Cl) and apparent volume of distribution (Vz) after a single i.v. dose of indicated bsTCE (0.1, 0.3 or 1.0 mg kg^{-1}) in NHP.

(G) Exemplifying gating strategy used to analyze T cell, monocyte, B cell and Vγ9-T cell frequencies and CD69 expression on Vγ9-T cells in NHP (here in peripheral blood).

For *in vivo*; n=1 per concentration per bsTCE. Negative control (NC) indicates PBS (A-C) or medium control (D-E). Box and whisker plots indicate the median, 25th-75th percentiles and minimum-maximum. Non-linear regression with 95% confidence bands (dotted lines) and EC_{50s} (dashed lines) (C,D). One-way ANOVA with Tukey multiple comparisons test (E).

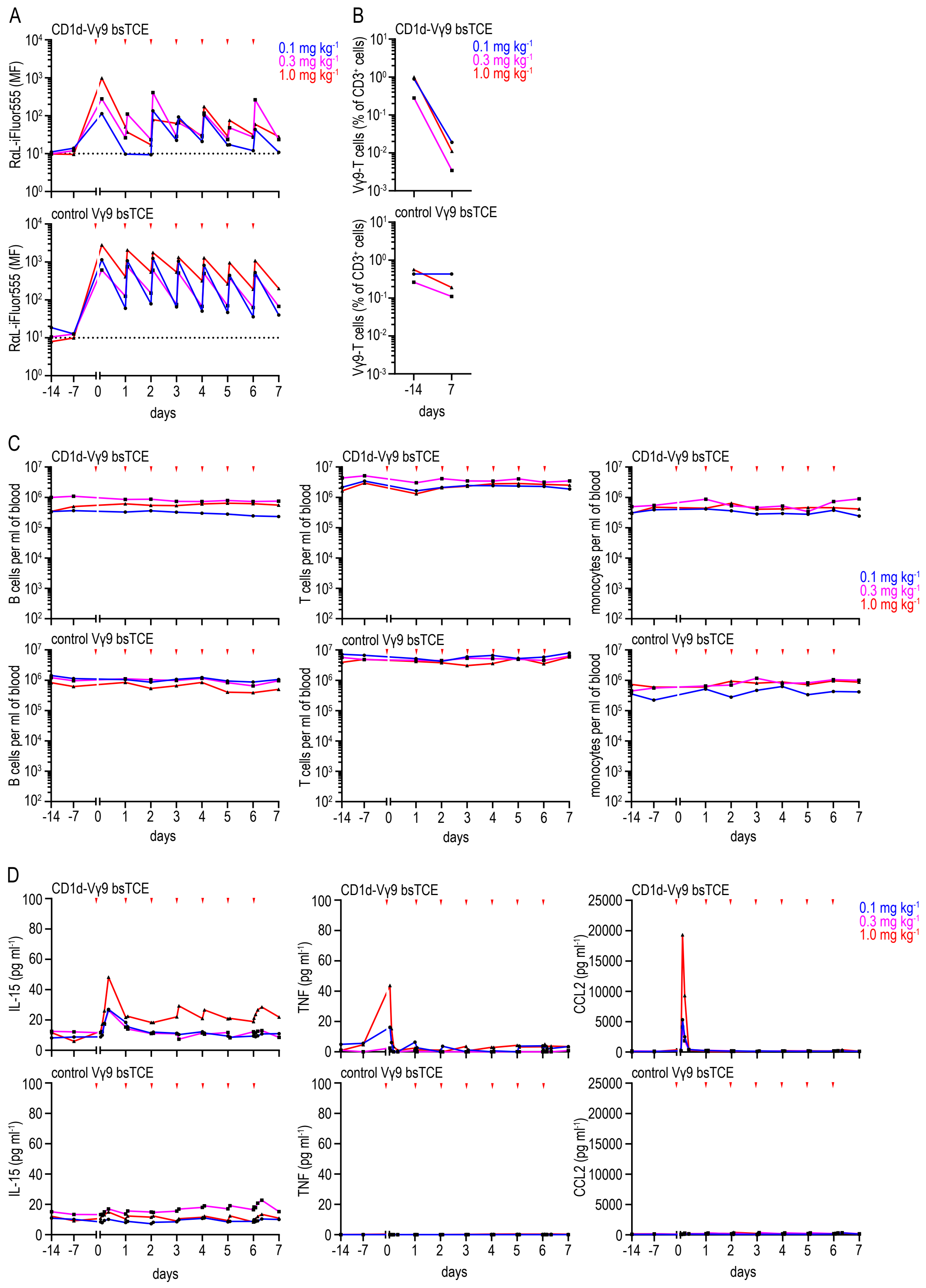
A
CD1d-Vγ9 bsTCE
control Vγ9 bsTCE
RαL-iFluor555 (MF)
0.1 mg kg-1
0.3 mg kg-1
1.0 mg kg-1
days
B
Vγ9-T cells (% of CD3+ cells)
C
B cells per ml of blood
T cells per ml of blood
monocytes per ml of blood
D
IL-15 (pg ml-1)
TNF (pg ml-1)
CCL2 (pg ml-1)

Figure S7. Infusion of CD1d-Vγ9 bsTCE does not affect B cell, T cell and monocyte frequencies (related to figure 7).

(A) Binding of indicated bsTCE to NHP Vγ9-T cells pre- and post-dose (2 and 24h, 7 daily doses, 0.1, 0.3 or 1.0 mg kg^{-1} i.v.), detected by rabbit-anti-llama (RαL)-iFluor555. MF, geometric mean fluorescence.

(B) NHP Vγ9-T cell percentage (of $CD3^+$ cells) in lymph node before dosing and after the last i.v. dose of indicated bsTCE (7 daily doses, 0.1, 0.3 or 1.0 mg kg^{-1} i.v.).

(C) NHP B cell, T cell and monocyte frequency over time before i.v. dosing (7 daily doses, 0.1, 0.3 or 1.0 mg kg^{-1} i.v.) of indicated bsTCE.

(D) Plasma cytokine levels pre- and post-dose (0.5, 2, 4, 8 and 24h after the first and last dose and 2 and 24h after the remaining doses, 0.1, 0.3 or 1.0 mg kg^{-1} i.v.) of indicated bsTCE.

Red arrowheads: CD1d-Vγ9 bsTCE or control Vγ9 bsTCE infusion. n=1 per concentration per bsTCE.

Table S1. Sequence alignment and CD1d-Vδ2 (hu-)bsTCE sequence (related to figure 1 and 4).

**Anti-CD1d**	**FR1**	**CDR1**	**FR2**	**CDR2**
VHH1D12.WT	QVQLVESGGGLVQAGGSLRLSCAAS	GSMFSDNV	MGWYRQAPGKQRELVAT	IRTGGST
VHH1D12.v1	**E**VQLVESGGGLVQ**P**GGSLRLSCAAS	GSMFSDNV	MGWYRQAPGKQRELVAT	IRTGGST
VHH1D12.v2	**E**VQLVESGGGLVQ**P**GGSLRLSCAAS	GSMFSDNV	MGWYRQAPGKQRELV**S**T	IRTGGST
VHH1D12.v3	**E**VQLVESGGGLVQ**P**GGSLRLSCAAS	GSMFSDNV	MGWYRQAPGKQRELV**S**T	IRTGGST
VHH1D12.v4	**E**VQLVESGGGLVQ**P**GGSLRLSCAAS	GSMFSDNV	M**S**WYRQAPGKQRELV**S**T	IRTGGST
VHH1D12.v5	**E**VQLVESGGGLVQ**P**GGSLRLSCAAS	GSMFSDNV	MGWYRQAPGK**GL**ELV**S**T	IRTGGST
VHH1D12.v6	**E**VQLVESGGGLVQ**P**GGSLRLSCAAS	GSMFSDNV	M**S**WYRQAPGK**GL**ELV**S**T	IRTGGST
VHH1D12.v7	**E**VQLVESGGGLVQ**P**GGSLRLSCAAS	GSMFSDNV	MGWYRQAPGK**ER**ELV**S**T	IRTGGST
VHH1D12.v8	**E**VQLVESGGG**S**VQ**P**GGSLRLSCAAS	GSMFSDNV	MGWYRQAPGK**ER**ELV**S**T	IRTGGST
VHH1D12.v9	**E**VQLVESGGGLVQ**P**GGSLRLSCAAS	GSMFSDNV	MGWYRQAPGK**GL**ELV**S**T	IRTGGST
VHH1D12.v10	**E**VQLVESGGG**S**VQ**P**GGSLRLSCAAS	GSMFSDNV	MGWYRQAPGK**GL**ELV**S**T	IRTGGST
VHH1D12.Q1E	**E**VQLVESGGGLVQAGGSLRLSCAAS	GSMFSDNV	MGWYRQAPGKQRELVAT	IRTGGST
**continued**	**FR3**		**CDR3**	**FR4**
VHH1D12.WT	NYADSVKGRFTISRDNAKNTVYLQMNSLKPEDTAVYYC		RHTIPVPSTPYDY	WGQGTQVTVSS
VHH1D12.v1	NYADSVKGRFTISRDNAKNTVYLQMNSL**RA**EDTAVYYC		RHTIPVPSTPYDY	WGQGTQVTVSS
VHH1D12.v2	NYADSVKGRFTISRDN**S**KNTVYLQMNSL**RA**EDTAVYYC		RHTIPVPSTPYDY	WGQGTQVTVSS
VHH1D12.v3	NYADSVKGRFTISRDN**S**KNT**L**YLQMNSL**RA**EDTAVYYC		RHTIPVPSTPYDY	WGQGT**L**VTVSS
VHH1D12.v4	NYADSVKGRFTISRDN**S**KNT**L**YLQMNSL**RA**EDTAVYYC		RHTIPVPSTPYDY	WGQGT**L**VTVSS
VHH1D12.v5	NYADSVKGRFTISRDN**S**KNT**L**YLQMNSL**RA**EDTAVYYC		RHTIPVPSTPYDY	WGQGT**L**VTVSS
VHH1D12.v6	NYADSVKGRFTISRDN**S**KNT**L**YLQMNSL**RA**EDTAVYYC		RHTIPVPSTPYDY	WGQGT**L**VTVSS
VHH1D12.v7	NYADSVKGRFTISRDN**S**KNTVYLQMNSL**RA**EDTAVYYC		RHTIPVPSTPYDY	WGQGTQVTVSS
VHH1D12.v8	NYADSVKGRFTISRDN**S**KNTVYLQMNSL**RA**EDTAVYYC		RHTIPVPSTPYDY	WGQGT**L**VTVSS
VHH1D12.v9	NYADSVKGRFTISRDN**S**KNTVYLQMNSL**RA**EDTAVYYC		RHTIPVPSTPYDY	WGQGT**L**VTVSS
VHH1D12.v10	NYADSVKGRFTISRDN**S**KNTVYLQMNSL**RA**EDTAVYYC		RHTIPVPSTPYDY	WGQGT**L**VTVSS
VHH1D12.Q1E	NYADSVKGRFTISRDNAKNTVYLQMNSLKPEDTAVYYC		RHTIPVPSTPYDY	WGQGTQVTVSS
**Anti-Vδ2**	**FR1**	**CDR1**	**FR2**	**CDR2**
VHH5C8.WT	EVQLVESGGGLVQAGGSLRLSCAAS	GRPFSNYA	MGWFRQAPGKEREFVAA	ISWSGGST
VHH5C8.v1	EVQL**L**ESGGG**S**VQ**P**GGSLRLSCAAS	GRPFSNYA	M**S**WFRQAPGKEREFV**S**A	ISWSGGST
VHH5C8.v2	EVQL**L**ESGGGLVQ**P**GGSLRLSCAAS	GRPFSNYA	M**S**WFRQAPGKEREFV**S**A	ISWSGGST
VHH5C8.v3	EVQL**L**ESGGG**S**VQ**P**GGSLRLSCAAS	GRPFSNYA	M**S**WFRQAPGK**GL**EFV**S**A	ISWSGGST
VHH5C8.v4	EVQL**L**ESGGGLVQ**P**GGSLRLSCAAS	GRPFSNYA	MGWFRQAPGKEREFVAA	ISWSGGST
VHH5C8.v5	EVQL**L**ESGGG**S**VQ**P**GGSLRLSCAAS	GRPFSNYA	MGWFRQAPGKEREFVAA	ISWSGGST
VHH5C8.v6	EVQL**L**ESGGGLVQ**P**GGSLRLSCAAS	GRPFSNYA	MGWFRQAPGKEREFV**S**A	ISWSGGST
VHH5C8.v7	EVQL**L**ESGGG**S**VQ**P**GGSLRLSCAAS	GRPFSNYA	MGWFRQAPGKEREFV**S**A	ISWSGGST
VHH5C8.v8	EVQL**L**ESGGGLVQ**P**GGSLRLSCAAS	GRPFSNYA	MGWFRQAPGKEREFV**S**A	ISWSGGST
VHH5C8.v9	EVQL**L**ESGGGLVQ**P**GGSLRLSCAAS	GRPFSNYA	MGWFR**E**APGKEREFV**S**A	ISWSGGST
VHH5C8.v10	EVQL**L**ESGGGLVQ**P**GGSLRLSCAAS	GRPFSNYA	MGWFR**E**APGKEREFV**S**A	ISWSGGST
**continued**	**FR3**		**CDR3**	**FR4**
VHH5C8.WT	SYADSVKGRFTISRDNAKNTVYLQMNSPKPEDTAIYYC		AAQFSGADYGFGRLGIRGYEYDY	WGQGTQVTVSS
VHH5C8.v1	SYADSVKGRFTISRDN**S**KNT**L**YLQMNS**LRA**EDTA**V**YYC		AAQFSGADYGFGRLGIRGYEYDY	WGQGTQVTVSS
VHH5C8.v2	SYADSVKGRFTISRDN**S**KNT**L**YLQMNS**LRA**EDTA**V**YYC		AAQFSGADYGFGRLGIRGYEYDY	WGQGT**L**VTVSS
VHH5C8.v3	SYADSVKGRFTISRDN**S**KNT**L**YLQMNS**LRA**EDTA**V**YYC		AAQFSGADYGFGRLGIRGYEYDY	WGQGT**L**VTVSS
VHH5C8.v4	SYADSVKGRFTISRDN**S**KNTVYLQMNS**LRA**EDTA**V**YYC		AAQFSGADYGFGRLGIRGYEYDY	WGQGT**L**VTVSS
VHH5C8.v5	SYADSVKGRFTISRDN**S**KNT**L**YLQMNS**LRA**EDTA**V**YYC		AAQFSGADYGFGRLGIRGYEYDY	WGQGT**L**VTVSS
VHH5C8.v6	SYADSVKGRFTISRDN**S**KNTVYLQMNS**LRA**EDTA**V**YYC		AAQFSGADYGFGRLGIRGYEYDY	WGQGT**L**VTVSS
VHH5C8.v7	SYADSVKGRFTISRDNAKNTVYLQMNS**LRA**EDTA**V**YYC		AAQFSGADYGFGRLGIRGYEYDY	WGQGT**L**VTVSS
VHH5C8.v8	SYADSVKGRFTISRDNAKNTVYLQMNS**LRA**EDTA**V**YYC		AAQFSGADYGFGRLGIRGYEYDY	WGQGT**L**VTVSS
VHH5C8.v9	SYADSVKGRFTISRDN**S**KNTVYLQMNS**LRA**EDTA**V**YYC		AAQFSGADYGFGRLGIRGYEYDY	WGQGT**L**VTVSS
VHH5C8.v10	SYADSVKGRFTISRDNAKNTVYLQMNS**LRA**EDTA**V**YYC		AAQFSGADYGFGRLGIRGYEYDY	WGQGT**L**VTVSS
CD1d-Vδ2 bsTCE	QVQLVESGGGLVQAGGSLRLSCAASGSMFSDNVMGWYRQAPGKQRELVATIRTGGSTNYADSVKGRFTISRDNAKNTVYLQMNSLKPEDTAVYYCRHTIPVPSTPYDYWGQGTQVTVSSGGGGSEVQLVESGGGLVQAGGSLRLSCAASGRPFSNYAMGWFRQAPGKEREFVAAISWSGGSTSYADSVKGRFTISRDNAKNTVYLQMNSPKPEDTAIYYCAAQFSGADYGFGRLGIRGYEYDYWGQGTQVTVSS			
CD1d-Vδ2 hu-bsTCE	**E**VQLVESGGGLVQAGGSLRLSCAASGSMFSDNVMGWYRQAPGKQRELVATIRTGGSTNYADSVKGRFTISRDNAKNTVYLQMNSLKPEDTAVYYCRHTIPVPSTPYDYWGQGTQVTVSSGGGGSEVQL**L**ESGGG**S**VQ**P**GGSLRLSCAASGRPFSNYAM**S**WFRQAPGKEREFV**S**AISWSGGSTSYADSVKGRFTISRDN**S**KNT**L**YLQMNS**LRA**EDTA**V**YYCAAQFSGADYGFGRLGIRGYEYDYWGQGTQVTVSS			

CDR according to IMGT numbering. Bold indicates amino acid substitutions.

Table S2. Humanization (related to figure 4).

	**Human sequence identity percentage (%)**	**HLA-DRB1-binding score**
**Anti-CD1d**	Ref. IGHV3-66*01	
VHH1D12.WT	76.3	523.5
VHH1D12.v1	80.4	398.9
VHH1D12.v2	82.5	386.4
VHH1D12.v3	83.5	332.1
VHH1D12.v4	84.5	346.7
VHH1D12.v5	85.6	346.4
VHH1D12.v6	86.6	361.0
VHH1D12.v7	82.5	349.0
VHH1D12.v8	81.4	297.1
VHH1D12.v9	84.5	346.4
VHH1D12.v10	83.5	348.8
VHH1D12.Q1E	77.3	523.5
**Anti-Vδ2**	Ref. IGHV3-23*01	
VHH5C8.WT	79.6	490.4
VHH5C8.v1	88.8	265.5
VHH5C8.v2	89.8	211.2
VHH5C8.v3	90.8	240.1
VHH5C8.v4	86.7	285.7
VHH5C8.v5	86.7	285.7
VHH5C8.v6	87.8	269.0
VHH5C8.v7	85.7	276.6
VHH5C8.v8	86.7	276.6
VHH5C8.v9	86.7	224.9
VHH5C8.v10	85.7	232.5

WT and humanized variant CD1d VHH1D12 and Vδ2 VHH5C8 sequence identity compared to the most similar human germline IGHV as indicated.

Table S3. Plasma cytokine levels in NHP pre- and post-dose bsTCE (related to figure 7).

Cytokine	Day	CD1d-Vγ9 bsTCE			control Vγ9 bsTCE		
		0.1 mg kg^{-1}	0.3 mg kg^{-1}	1.0 mg kg^{-1}	0.1 mg kg^{-1}	0.3 mg kg^{-1}	1.0 mg kg^{-1}
**IL-1β**	-14	1.63	0.08	0.21	0.13	0.13	2.25
	-7	1.98	0.05	1.77	0.13	0.13	2.33
	0						
	0 + 0.5h	0.05	0.05	1.68	0.13	1.17	2.42
	0 + 2h	1.05	0.05	0.82	0.13	0.56	2.39
	0 + 4h	0.05	0.05	0.96	0.13	0.68	3.22
	0 + 8h	0.05	0.05	0.05	0.13	0.74	2.36
	1	1.95	0.05	0.81	0.13	0.13	1.65
	1 + 2h	0.45	0.05	0.36	0.13	0.13	0.81
	2	0.05	0.05	0.18	1.29	0.13	1.98
	2 + 2h	0.49	0.05	0.05	1.23	0.13	2.11
	3	0.05	0.05	0.93	0.96	0.13	0.13
	3 + 2h	0.05	0.05	0.05	2.05	0.13	0.13
	4	0.05	0.05	0.79	0.13	0.13	2.15
	4 + 2h	0.28	0.05	0.99	0.49	0.13	2.2
	5	0.17	0.05	1.07	0.13	0.13	3.72
	5 + 2h	1.42	0.05	1.25	0.67	0.13	1.2
	6	1.1	0.05	0.72	0.13	0.13	1.81
	6 + 0.5h	0.23	0.05	0.05	0.27	0.13	2.22
	6 + 2h	1.3	0.05	1.67	0.13	1.87	1.68
	6 + 4h	0.64	0.05	0.98	0.13	1.76	2.39
	6 + 8h	0.48	0.05	1.31	0.13	1.98	4.63
	7	1.22	0.05	1.32	0.13	1.91	3.73
**IL-4**	-14	0.74	0.16	0.15	0.13	0.13	2.25
	-7	0.87	0.04	0.57	0.13	0.13	2.33
	0						
	0 + 0.5h	0.01	0.09	0.57	0.13	1.17	2.42
	0 + 2h	0.73	0.11	0.25	0.13	0.56	2.39
	0 + 4h	0.01	0.1	0.35	0.13	0.68	3.22
	0 + 8h	0.01	0.12	0.11	0.13	0.74	2.36
	1	0.67	0.18	0.26	0.13	0.13	1.65
	1 + 2h	0.22	0.04	0.19	0.13	0.13	0.81
	2	0.01	0.01	0.11	1.29	0.13	1.98
	2 + 2h	0.55	0.01	0.13	1.23	0.13	2.11
	3	0.01	0.09	0.43	0.96	0.13	0.13
	3 + 2h	0.27	0.01	0.09	2.05	0.13	0.13
	4	0.01	0.01	0.39	0.13	0.13	2.15
	4 + 2h	0.16	0.01	0.45	0.49	0.13	2.2
	5	0.06	0.06	0.45	0.13	0.13	3.72
	5 + 2h	0.46	0.01	0.46	0.67	0.13	1.2
	6	0.46	0.13	0.37	0.13	0.13	1.81
	6 + 0.5h	0.19	0.09	0.15	0.27	0.13	2.22
	6 + 2h	0.37	0.06	0.57	0.13	1.87	1.68
	6 + 4h	0.18	0.09	0.48	0.13	1.76	2.39
	6 + 8h	0.2	0.05	0.53	0.13	1.98	4.63
	7	0.42	0.21	0.55	0.13	1.91	3.73
**IL-8**	-14	2.45	0.47	0.14	0.16	0.15	0.09

	-7	2.63	0.01	1.75	0.03	0.2	0.16
	0						
	0 + 0.5h	0.18	0.03	1.21	0.17	0.11	0.03
	0 + 2h	2.08	0.23	1.07	0.2	0.03	0.03
	0 + 4h	0.03	0.12	0.87	0.07	0.06	0.08
	0 + 8h	0.03	0.03	0.03	0.15	0.12	0.03
	1	2.21	0.41	0.86	0.07	0.11	0.03
	1 + 2h	1.46	0.03	0.54	0.11	0.11	0.03
	2	0.03	0.05	0.15	0.06	0.03	0.03
	2 + 2h	1.67	0.03	0.03	0.1	0.11	0.03
	3	0.03	0.03	1.32	0.18	0.03	0.13
	3 + 2h	1.04	0.03	0.19	0.19	0.04	0.12
	4	0.14	0.07	0.89	0.07	0.03	0.15
	4 + 2h	0.38	0.03	0.95	0.24	0.06	0.13
	5	0.61	0.03	1.51	0.03	0.03	0.2
	5 + 2h	1.51	0.03	1.13	0.08	0.09	0.15
	6	1.44	0.16	1.06	0.05	0.09	0.06
	6 + 0.5h	0.45	0.03	0.42	0.06	0.11	0.16
	6 + 2h	1.36	0.03	1.77	0.1	0.08	0.13
	6 + 4h	0.56	0.03	1.47	0.09	0.03	0.24
	6 + 8h	0.64	0.03	1.92	0.03	0.07	0.22
	7	1.06	0.37	1.17	0.11	0.1	0.19
**IL-10**	-14	1.9	0.15	0.17	0.17	0.01	0.15
	-7	2.35	0.01	1.25	0.2	0.12	0.14
	0						
	0 + 0.5h	0.15	0.01	1.37	0.01	0.1	0.01
	0 + 2h	1.91	0.11	0.4	0.01	0.06	0.12
	0 + 4h	0.01	0.01	0.59	0.09	0.1	0.15
	0 + 8h	0.01	0.01	0.11	0.06	0.01	0.07
	1	1.82	0.28	0.59	0.08	0.05	0.01
	1 + 2h	1.13	0.01	0.29	0.01	0.01	0.06
	2	0.01	0.01	0.27	0.13	0.01	0.04
	2 + 2h	1.67	0.01	0.15	0.19	0.07	0.1
	3	0.01	0.22	0.81	0.16	0.12	0.01
	3 + 2h	0.71	0.01	0.01	0.16	0.06	0.17
	4	0.05	0.01	0.84	0.21	0.01	0.09
	4 + 2h	0.44	0.01	1.02	0.21	0.08	0.12
	5	0.29	0.01	0.93	0.01	0.09	0.07
	5 + 2h	1.26	0.01	0.75	0.01	0.09	0.16
	6	1.27	0.01	0.74	0.03	0.01	0.21
	6 + 0.5h	0.42	0.01	0.12	0.01	0.01	0.01
	6 + 2h	1.2	0.01	1.07	0.01	0.01	0.1
	6 + 4h	0.59	0.01	0.75	0.01	0.01	0.12
	6 + 8h	0.6	0.01	0.94	0.01	0.12	0.14
	7	1.08	0.37	0.88	0.01	0.08	0.21
**IL-12p70**	-14	12.36	0.03	1.96	0.03	0.81	0.69
	-7	14.48	0.03	11.57	0.21	1.74	0.83
	0						
	0 + 0.5h	0.82	0.03	11.65	0.03	0.62	0.55
	0 + 2h	10.25	0.28	5.5	0.03	0.83	0.03

	0 + 4h	0.76	0.03	7.79	0.03	1.02	0.67
	0 + 8h	0.03	0.03	0.99	0.03	0.7	0.45
	1	11.86	0.03	5.47	0.03	0.68	0.3
	1 + 2h	5.5	0.03	2.84	0.03	0.66	0.38
	2	0.03	0.03	1.7	0.17	1.13	0.17
	2 + 2h	11	0.03	1.06	0.03	0.48	0.03
	3	0.03	0.03	6.53	0.03	1.1	0.64
	3 + 2h	6.81	0.03	0.66	0.03	0.62	0.48
	4	0.66	0.03	5.77	0.2	0.64	0.37
	4 + 2h	4.38	0.03	6.76	0.29	0.68	0.71
	5	0.64	0.03	7.26	0.26	0.58	0.61
	5 + 2h	8.57	0.03	5.95	0.03	0.54	0.79
	6	8.65	0.03	5.14	0.08	0.61	0.56
	6 + 0.5h	2.22	0.03	1.28	0.03	0.8	0.53
	6 + 2h	8.24	0.03	9.31	0.03	0.31	0.68
	6 + 4h	3.4	0.03	5.97	0.16	0.48	0.54
	6 + 8h	4.28	0.03	6.68	0.3	1.09	0.4
	7	8.99	0.03	7.47	0.03	0.91	0.36
**IFN-γ**	-14	26.08	0.13	0.13	0.13	0.13	2.25
	-7	33.65	0.13	14.33	0.13	0.13	2.33
	0						
	0 + 0.5h	0.13	0.13	12.21	0.13	1.17	2.42
	0 + 2h	23.98	0.13	6.51	0.13	0.56	2.39
	0 + 4h	0.13	0.13	9.64	0.13	0.68	3.22
	0 + 8h	0.13	0.13	0.13	0.13	0.74	2.36
	1	24.44	0.13	6.4	0.13	0.13	1.65
	1 + 2h	5.83	0.13	0.13	0.13	0.13	0.81
	2	0.13	0.13	0.13	1.29	0.13	1.98
	2 + 2h	32.43	0.13	0.13	1.23	0.13	2.11
	3	0.13	0.13	10.29	0.96	0.13	0.13
	3 + 2h	6.81	0.13	3.39	2.05	0.13	0.13
	4	0.13	0.13	12.67	0.13	0.13	2.15
	4 + 2h	7.96	0.13	14.64	0.49	0.13	2.2
	5	1.91	0.13	12.89	0.13	0.13	3.72
	5 + 2h	15.02	0.13	10.24	0.67	0.13	1.2
	6	15.02	0.13	7.99	0.13	0.13	1.81
	6 + 0.5h	4.58	0.13	0.13	0.27	0.13	2.22
	6 + 2h	12.67	0.13	18.17	0.13	1.87	1.68
	6 + 4h	4.5	0.13	14.11	0.13	1.76	2.39
	6 + 8h	6.59	0.13	14.72	0.13	1.98	4.63
	7	12.67	0.13	15.15	0.13	1.91	3.73

Plasma cytokine levels pre- and post-dose (0.5, 2, 4, 8 and 24h after the first and last dose and 2 and 24h after the remaining doses, 0.1, 0.3 or 1.0 mg kg^{-1}) of indicated bsTCE.

Table S4. Clinical, hematological and biochemical parameters in NHP pre- and post-dose bsTCE (related to figure 7).

	Day	CD1d-Vγ9 bsTCE			control Vγ9 bsTCE		
		0.1 mg kg^{-1}	0.3 mg kg^{-1}	1.0 mg kg^{-1}	0.1 mg kg^{-1}	0.3 mg kg^{-1}	1.0 mg kg^{-1}
**Body weight (kg)**	-14	4	4.4	4.7	4.5	4.5	5.2
	-7	3.9	4.4	4.7	4.5	4.5	5.2
	0	4.1	4.5	4.6	4.4	4.5	5.1
	7	4.1	4.4	4.5	4.4	4.5	5.1
**RBC (x10^{12} l^{-1})**	-14	6.66	6.14	6.44	5.98	6.43	6.04
	-7	6.44	6.01	6.03	5.62	6	5.57
	1	6.26	5.98	5.74	5.5	5.37	5.27
	2	5.98	5.63	5.47	5.4	5.27	5.11
	3	5.73	5.41	5.63	5.18	5.21	4.91
	4	5.24	5.11	5.46	5.03	4.94	4.5
	5	5.06	4.81	4.73	5.1	4.84	4.52
	6	5.06	4.49	4.48	4.71	4.65	4.44
	7	4.84	4.34	4.34	4.71	4.44	4.22
**PLT (x10^{9} l^{-1})**	-14	573	558	428	308	533	389
	-7	558	465	486	303	538	450
	1	569	360	391	341	532	439
	2	555	367	388	263	496	424
	3	583	393	424	315	485	408
	4	591	404	437	286	447	400
	5	682	448	433	253	476	418
	6	623	465	424	247	475	463
	7	672	458	440	309	528	462
**PT (s)**	-14	9.2	9.2	8.9	8.9	9.1	9.1
	-7	8.9	9.1	8.9	8.8	9	9.3
	3	9	9.3	9.4	8.9	9.6	9.8
	7	9.2	9.3	9.5	8.9	9.8	9.3
**Total bilirubin (µmol l^{-1})**	-14	5.1	2	2.4	3.4	3.2	2.3
	-7	3.6	2.3	2.9	3.6	2.4	2.1
	2	4	2.6	2.7	4.7	2.4	2.5
	5	3.4	3	3.8	3.4	1.9	2.7
	7	3.2	3.3	3.5	3.3	2.1	3.5
**ALT (U l^{-1})**	-14	47	153.7	52.4	76.8	70.9	33.8
	-7	69.6	132	61.1	34.4	78.1	29.4
	2	62.9	140.6	51.2	29.9	71.3	38
	5	48.4	135.4	62.9	25.5	60.3	43.8
	7	39.4	111.6	68	28.7	53.7	41.5
**Creatinine (µmol l^{-1})**	-14	63	63	66	71	47	69
	-7	59	59	74	72	46	65
	2	55	57	68	72	43	61
	5	55	56	66	75	46	64
	7	57	53	65	73	45	64

Clinical, hematological and biochemical parameters pre- and post-dose (0.1, 0.3 or 1.0 mg kg^{-1}) of indicated bsTCE. RBC, red blood cells. PLT, platelets. PT, prothrombin time. ALT, alanine aminotransferase.
